# New derivatives of 6-nitrocoumarin-3-thiosemicarbazone exhibit improved antiparasitic activity in the placenta against *Trypanosoma cruzi* and *Toxoplasma gondii*

**DOI:** 10.1128/aac.00454-25

**Published:** 2025-08-07

**Authors:** Esteban Rocha-Valderrama, Santiago Rostán, Mercedes Fernández, Ana Liempi, Christian Castillo, Graciela Mahler, Ambar Galarza-Jarrin, Jaime A. Costales, Josué Pozo-Martínez, Claudio Olea-Azar, Lucía Otero, Mauricio Moncada-Basualto

**Affiliations:** 1 Instituto Universitario de Investigación y Desarrollo Tecnológico, Universidad Tecnológica Metropolitana 28092, Santiago, Chile; 2 Inorganic and Analytical Department, Free Radical and Antioxidants Laboratory, Faculty of Chemical and Pharmaceutical Sciences, University of Chile 14655, Santiago, Chile; 3 Área Química Inorgánica, DEC, Facultad de Química, Universidad de la República 201894 , Montevideo, Uruguay; 4 Graduate Program in Chemistry, Facultad de Química, Universidad de la República 201894 , Montevideo, Uruguay; 5 Programa de Biología Integrativa, Instituto de Ciencias Biomédicas, Facultad de Medicina, Universidad de Chile, Santiago, Chile; 6 Departamento de Química Orgánica, Laboratorio de Química Farmacéutica, Facultad de Química, Universidad de la República 201894 , Montevideo, Uruguay; 7 Centro de Investigación para la Salud en América Latina, Pontificia Universidad Católica del Ecuador 27884, Quito, Pichincha, Ecuador; 8 Laboratorio de Química-Médica, Facultad de Ciencia y Tecnología, Universidad del Azuay 27892, Cuenca, Azuay, Ecuador; The Children's Hospital of Philadelphia, Philadelphia, Pennsylvania, USA

**Keywords:** 6-nitrocoumarin-3-thiosemicarbazone, *Trypanosoma cruzi*, *Toxoplasma gondii*, *in vitro* and *ex vivo* models

## Abstract

Congenital infections by *Trypanosoma cruzi* and *Toxoplasma gondii* pose significant clinical challenges due to the lack of safe and effective treatments. This study evaluates eight novel 6-nitrocoumarin-3-thiosemicarbazone derivatives in an *ex vivo* human placenta model, assessing their antiparasitic activity and impact on tissue integrity. Two therapeutic approaches were tested: pre-infection (preventive) and post-infection (therapeutic). *In vitro* and *ex vivo* assays revealed strong activity trends. Compound **7** was the most effective against *T. cruzi* (IC_50_ = 22.4 ± 0.8 µM, logP = 2.49), while compound **1** exhibited the highest activity against *T. gondii* (IC_50_ = 17.3 ± 0.5 µM, logP = 1.44). Unlike current treatments, none of the compounds induced placental tissue damage, preserving trophoblast function. Structure-activity relationship (SAR) analysis identified an inverse correlation between lipophilicity and antiparasitic activity in *T. gondii*, where polar compounds were more effective. In *T. cruzi*, higher lipophilicity favored trypanocidal activity, suggesting differential cell permeability mechanisms. Mechanistic studies using electrochemistry and electron spin resonance (ESR) demonstrated that nitro group bioreduction promotes ROS generation, explaining activity against *T. cruzi*. By contrast, lower ROS levels in *T. gondii* suggest alternative mechanisms. This study validates the *ex vivo* human placenta model as a clinically relevant platform for antiparasitic drug screening. The findings highlight 6-nitrocoumarin-3-thiosemicarbazones as promising early-stage candidates that warrant further optimization to develop safer and more effective therapies for congenital infections.

## INTRODUCTION

Zoonotic infections caused by protozoa, such as *Trypanosoma cruzi* and *Toxoplasma gondii*, represent important public health problems due to their high morbidity, socioeconomic impact, and the limitations of current treatments. Congenital transmission of both diseases poses a significant challenge, as it affects vulnerable populations such as newborns and their mothers, generating severe complications for both neonatal development and maternal health (1, 2). Chagas disease, caused by *T. cruzi*, affects more than 6 million people in Latin America, with 70 million individuals at risk of infection. Although traditionally considered an endemic disease, its prevalence has increased in non-endemic regions due to factors such as migration and congenital transmission, the latter responsible for a considerable proportion of cases in developed countries (3).

On the other hand, toxoplasmosis, caused by *T. gondii*, affects 30% of the world’s population, with a significant clinical impact on immunocompromised individuals and pregnant women. Vertical transmission of *T. gondii* can cause spontaneous abortion, congenital malformations, and severe neurological sequelae in the newborn (4). Both infections share complex transmission mechanisms involving interactions between the parasite, the placental barrier, and maternal-fetal immune responses (5, 6). In this context, human placental explant (HPE) models have emerged as valuable tools to study tissue infection, assess damage to the placental barrier, and analyze the effect of new compounds on placental tissue (7, 8).

**Fig 1:**
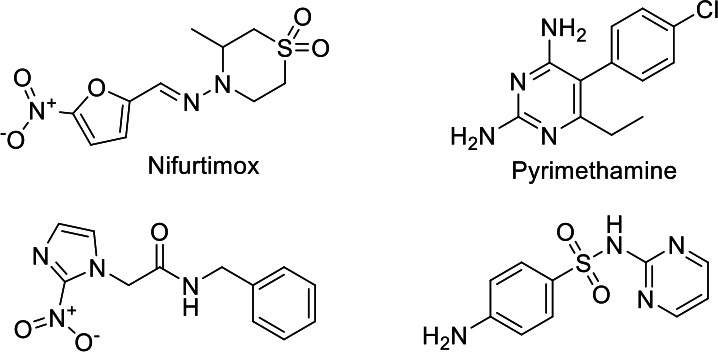
Drugs in use for the treatment of *Trypanosoma cruzi* and *Toxoplasma gondii* infections.

Current treatments for Chagas disease and toxoplasmosis have significant limitations in efficacy and safety, particularly during pregnancy. In the case of *T. cruzi*, the nitro compounds benznidazole (BNZ) and nifurtimox (NFX) are the main therapeutic options (Fig. 1). These act as prodrugs that generate reactive oxygen species (ROS), inducing oxidative stress in the parasite. However, their efficacy is limited to the acute phase of the disease, and they have significant adverse effects, including teratogenicity, which restricts their use during pregnancy (9–12). For *T. gondii*, the combination of pyrimethamine (PMN) and sulfadiazine (SDZ) is the treatment of choice (13). However, its antifolate activity also affects folate metabolism in humans, limiting its use in the first trimester of pregnancy due to the risk of defects in the development of the fetus’s central nervous system (14). Therefore, there is a need for new compounds with selective antiparasitic activity.

Rojo et al. characterized the differences in the efficacy of NFX and BNZ in HPE infected with *T. cruzi* (7). Although neither drug completely eradicated the parasitic DNA load under the conditions evaluated, NFX was observed to induce inherent toxicity and tissue damage, possibly related to its mechanism of action. *T. cruzi* displays high genetic variability, and seven major genetic lineages, termed discrete typing units (DTUs), are recognized (15, 16). These DTUs (TcI-TcVI and Tcbat) are unevenly distributed throughout endemic regions in Latin America. Although no proven correlation exists between *T. cruzi* DTUs and drug sensitivity/effectiveness of treatment, wide variations in susceptibility occur among *T. cruzi* strains belonging to the same DTU in terms of drug resistance (17).

Regarding the reference drugs for *T. gondii*, PMN, particularly in combination with sulfadiazine, effectively reduces the infection and proliferation of *T. gondii* in HPE. However, PMN has demonstrated toxicity against trophoblastic cells of the BeWo cell line (18). Therefore, employing histological and histochemical analyses to evaluate possible tissue damage of new antiparasitic compounds against *T. cruzi* and *T. gondii* is helpful. This approach not only allows for the assessment of the pharmacological effects of the compounds in this biological model but also helps to verify any intrinsic damage these compounds may cause.

The search and design of new compounds with antiparasitic activity have been based on therapeutic targets present in the parasites, absent in the host, or structurally different from those in the host (19). In this sense, thiosemicarbazones have been reported as molecules with important antiparasitic activity, and several derivatives of these have been evaluated against both *T. cruzi* and *T. gondii* (20–22). The mechanism of action of anti-*T*. *gondii* of these compounds is not precisely known. However, recent studies have suggested that its effect may be due to interference with protein biosynthesis, leading to mitochondrial damage and alterations of the parasitophorous vacuole (23).

**Fig 2:**
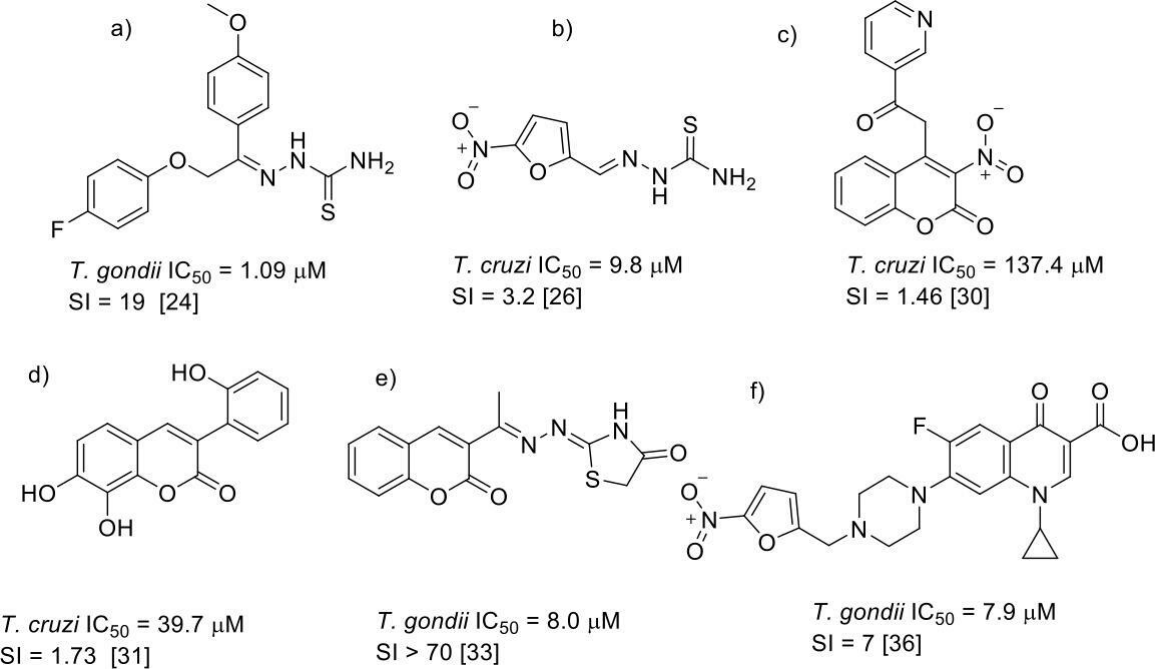
Representative thiosemicarbazone (a–b, e) and coumarin (c–f) derivatives with activity against *T. cruzi* or *T. gondii*. Compound (a) shows potent anti-*T. gondii* activity; (b) is a nitrofuran–thiosemicarbazone with activity against *T. cruzi*; (c–d) are coumarins evaluated as trypanocidal agents; (e) is a highly selective coumarin–thiosemicarbazone hybrid; and (f) is a nitrofuran–quinolone–coumarin hybrid active against *T. gondii*. SI = EC₅₀ (mammalian cell model) / IC₅₀ (parasite).

In addition, computational studies have demonstrated that these structures can potently inhibit certain cathepsins, which are important virulence factors in the cell invasion process of *T. gondii* (24, 25). An example of a thiosemicarbazone derivative with activity against *T. gondii* is shown in Fig. 2 (compound a) (26). On the other hand, the mechanism of action of thiosemicarbazone derivatives against *T. cruzi* has been related to inhibiting the parasite’s main cysteine protease, cruzipain, which is crucial in the parasite’s infection of cells and tissues (27). We had previously reported the anti-*T*. *cruzi* activity of nitrofuryl-containing thiosemicarbazones (Fig. 2, compound b), in which trypanocidal activity was associated with the generation of oxidative stress through the bioreduction of the nitro moiety as it has been described for NFX (28).

Coumarins have been described as molecules with diverse biological activities, including anticoagulant, antioxidant, and antiparasitic activity, and they are being evaluated as trypanocidal and antimalarial compounds (29–31). Against *T. cruzi*, coumarin derivatives have shown antiparasitic activity, possibly associated with oxidative stress due to imbalances in the mitochondrial membrane potential and the possible inhibition of the parasite’s glycosomal glyceraldehyde 3-phosphate dehydrogenase (gGAPDH), structurally different from human GAPDH. Some examples are shown in Fig. 2 (compounds c and d). Recent studies have shown that the addition of nitro groups to coumarins significantly improves their activity due to the generation of ROS mediated by the reduction of the nitro group (32, 33). Coumarin’s activity against *T. gondii* has been assessed in a few instances, and they are being used as substituents for compounds that have demonstrated anti-*T*. *gondii* activity. The works described by D'Ascenzio et al. (34) (Fig. 2, compound e) and Chimenti et al. (35) indicate that the addition of a coumarin structure to the basic skeleton of thiazole derivatives increases toxoplasmicidal activity and selectivity against this parasite. Molina et al. (36) described that 4-thiazolidinone derivatives inhibit the activity of protein kinase 1, altering the infective and reproductive capacity of the parasite. Likewise, the high activity of quinolone-coumarin hybrid compounds has been described (37). In addition, nitrofurans linked to quinolones have shown high anti-*T*. *gondii* activity (Fig. 2, compound f) (38).

Based on the above, including coumarin and thiosemicarbazone moieties in a single molecule could be a good strategy to obtain compounds with improved anti-*T*. *cruzi* and anti-*T*. *gondii* activity and selectivity. In this sense, we had developed new protocols for the preparation of a series of coumarin-thiosemicarbazone hybrid compounds (39). The prepared compounds were evaluated *in vitro* against the trypomastigote form of *T. cruzi* (Dm28c strain), but most showed negligible activity (40, 41). Recently, Alves Nunes et al. studied the activity of a series of coumarin-3-thiosemicarbazone hybrids with substituents in the coumarin fraction and without substituents in the thiosemicarbazone fraction (42). Their study primarily focused on enzymatic inhibition, particularly cruzipain, reporting high inhibitory activity with IC_50_ values in the nanomolar range. However, this potent enzymatic inhibition did not translate into strong antiparasitic effects in *T. cruzi*, with IC_50_ values not significantly improved compared to reference drugs. However, this suggests their rational design can optimize antiparasitic activity.

**Fig 3:**
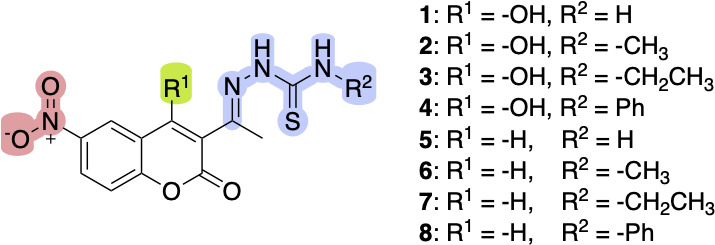
6-Nitrocoumarin-3-thiosemicarbazone hybrids 1–8.

As a strategy for improving the antiparasitic activity of the coumarin-thiosemicarbazone hybrid compounds, a nitro group could be included as a substituent in the coumarin ring. This nitro moiety could be involved in the generation of oxidative stress in the parasites, leading to an enhanced antiparasitic activity, as it has been previously described for other nitro compounds (28). This study synthesized eight new 6-nitrocoumarin-3-thiosemicarbazone hybrid compounds (**1–8**), with seven being reported for the first time and fully characterized. The prepared compounds differ by having a hydroxyl group at the 4-position of the coumarin moiety and include substituents with varying lipophilicity in the thiosemicarbazone part (Fig. 3). To evaluate their therapeutic potential, these compounds were thoroughly investigated in *in vitro* and *ex vivo* models relevant to congenital infections, focusing on their effects on *T. cruzi* and *T. gondii* in infected cells and placental tissue. In addition, assessment in human placental explants (HPEs) enabled the analysis of antiparasitic activity in a physiologically relevant setting, providing insights into the compounds' ability to decrease parasitic load without causing noticeable alterations in tissue architecture. The correlation between *in vitro* and ex vivo activity highlights the significance of these models in predicting the therapeutic potential of new agents. Moreover, the relationship between the electrochemical reduction of the nitro group and the formation of reactive oxygen species (ROS) indicates a potential mechanism of action that was investigated in this study.

## RESULTS AND DISCUSSION

### Study design

The development of 6-nitrocoumarin-3-thiosemicarbazone hybrids stems from our group’s continuous interest in exploring the chemical and biological potential of coumarin and thiosemicarbazone derivatives as antiparasitic agents. Previous studies conducted in our laboratory and research from other groups have shown that thiosemicarbazones exhibit significant trypanocidal activity, primarily mediated by cruzipain inhibition and the induction of oxidative stress (32, 42). In addition, coumarins have demonstrated remarkable potential as inhibitors of key enzymes involved in the metabolism of *T. cruzi* and *T. gondii*. This provides a basis for the rational design of hybrid molecules that combine the antiparasitic properties of both chemical scaffolds (43, 44).

Our previous work reported the synthesis and characterization of coumarin-3-thiosemicarbazone hybrids, highlighting their structural diversity and ability to generate reactive oxygen species (ROS) (39–41). However, although these compounds exhibited activity against *T. cruzi*, their efficacy against the infective forms of the parasite was limited. These findings suggest that while the non-nitro compounds showed some ability to inhibit parasite proliferation, structural optimization was necessary to enhance their efficacy. In this regard, our previous studies emphasized the importance of structural modifications, such as the incorporation of nitro groups, in improving the biological activity of indazolin-3-ones (45). Electrochemical analyses further confirmed this strategy, which demonstrated that nitro-functionalized derivatives can enhance ROS production, a critical factor for their antiparasitic efficacy. Moreover, computational and spectroscopic studies provided crucial insights into the Z/E isomerization equilibria of these hybrids (39).

Given this background, the present study aims to broaden the scope of our previous research by synthesizing a new series of 6-nitrocoumarin-3-thiosemicarbazone derivatives with varying degrees of lipophilicity. The design of these compounds focuses on optimizing their lipophilicity to enhance membrane permeability and, consequently, improve their antiparasitic activity. The strategic incorporation of functional groups such as hydroxyl and nitro was proposed to investigate their influence on these hybrids’ redox behavior and biological properties. Prior studies informed the introduction of the nitro group at the 6-position on 3-nitrocoumarins, which demonstrated that the positioning of the nitro group within the coumarin structure significantly affects ROS generation and antiparasitic activity (32). The 6-position was chosen under the hypothesis that it could increase the stabilization of the radical generated during nitro group reduction, thereby enhancing biological activity compared to compounds with the nitro group at the 3-position, where interference from the 4-position substituent hinders efficient delocalization of the unpaired electron, reducing ROS generation and antiparasitic efficacy.

Since the antiparasitic effect of these compounds depends on their chemical structure and ability to function in a complex biological environment, we assessed their impact using both cellular and infection models. Based on the evidence regarding cruzipain inhibition (42), we decided to evaluate the antiparasitic activity of these compounds and their effects on tissue infection under two experimental conditions: co-incubation with parasites and post-infection. This evaluation was essential for determining how the compounds influence parasite load in a more physiological context using an *ex vivo* model with human placental explants. This approach allowed us to analyze their effects on extracellular and intracellular parasite forms in a setting that closely resembles the biological reality of infection, providing a solid foundation for future structural optimizations.

### Chemistry

**Fig 4:**
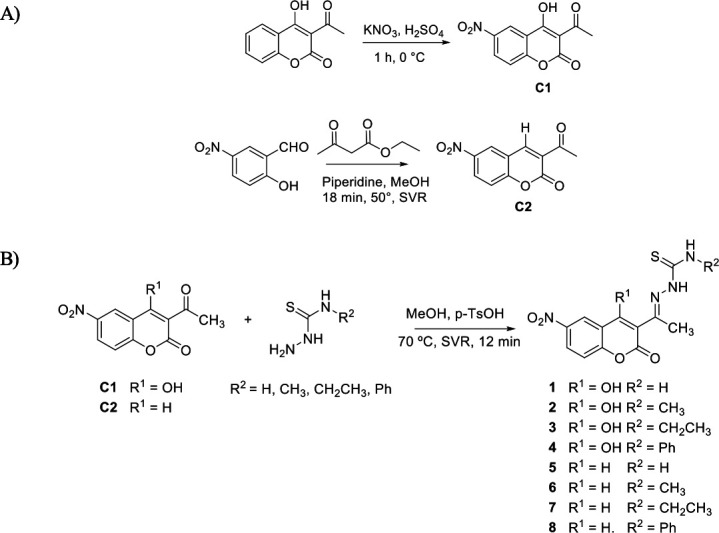
(A) Synthesis of coumarins C1 and C2; (B) synthesis of compounds 1–8.

Compounds **1–4** and **5–8** were obtained by the condensation of the corresponding thiosemicarbazide derivatives with 6-nitro-4-hydroxy-3-acetyl-coumarin (**C1**) or 6-nitro-3-acetyl-coumarin (**C2**), respectively (Fig. 4), using a sealed-vessel reactor (SVR), a methodology previously developed by us for similar compounds (39). The starting coumarins **C1** and **C2** were prepared, as shown in Fig. 4A. **C1** was prepared from 4-hydroxy-3-acetylcoumarin by nitration reaction with H_2_SO_4_ and KNO_3_ (46), while **C2** was prepared by a Knoevenagel reaction between 5-nitrosalicylaldehyde and ethyl acetoacetate (47). All nitrocoumarin-thiosemicarbazone derivatives were obtained in moderate to very good yields (60%–86%). They were spectroscopically characterized in solid state by FTIR (see Table S1 at https://doi.org/10.5281/zenodo.15694236) and in solution by ^1^H- and ^13^C-NMR (see Tables S2 and S3 at https://doi.org/10.5281/zenodo.15694236).

### Electrochemical characterization

As previously stated, the potential mode of antiparasitic action of the prepared nitro compounds could be related to the generation of oxidative stress through the bioreduction of the nitro moiety, generating free radical species involved in oxidative stress (28). To assess the potential of compounds **1–8** for having this mode of action, their electrochemical characterization in the cathodic direction was performed at room temperature by cyclic voltammetry (CV) in DMSO solutions using a hanging drop mercury electrode (HDME). The electrochemical behavior of these compounds provides insight into their potential bioactivation and ROS generation, which are critical factors in their antiparasitic efficacy.

**Fig 5:**
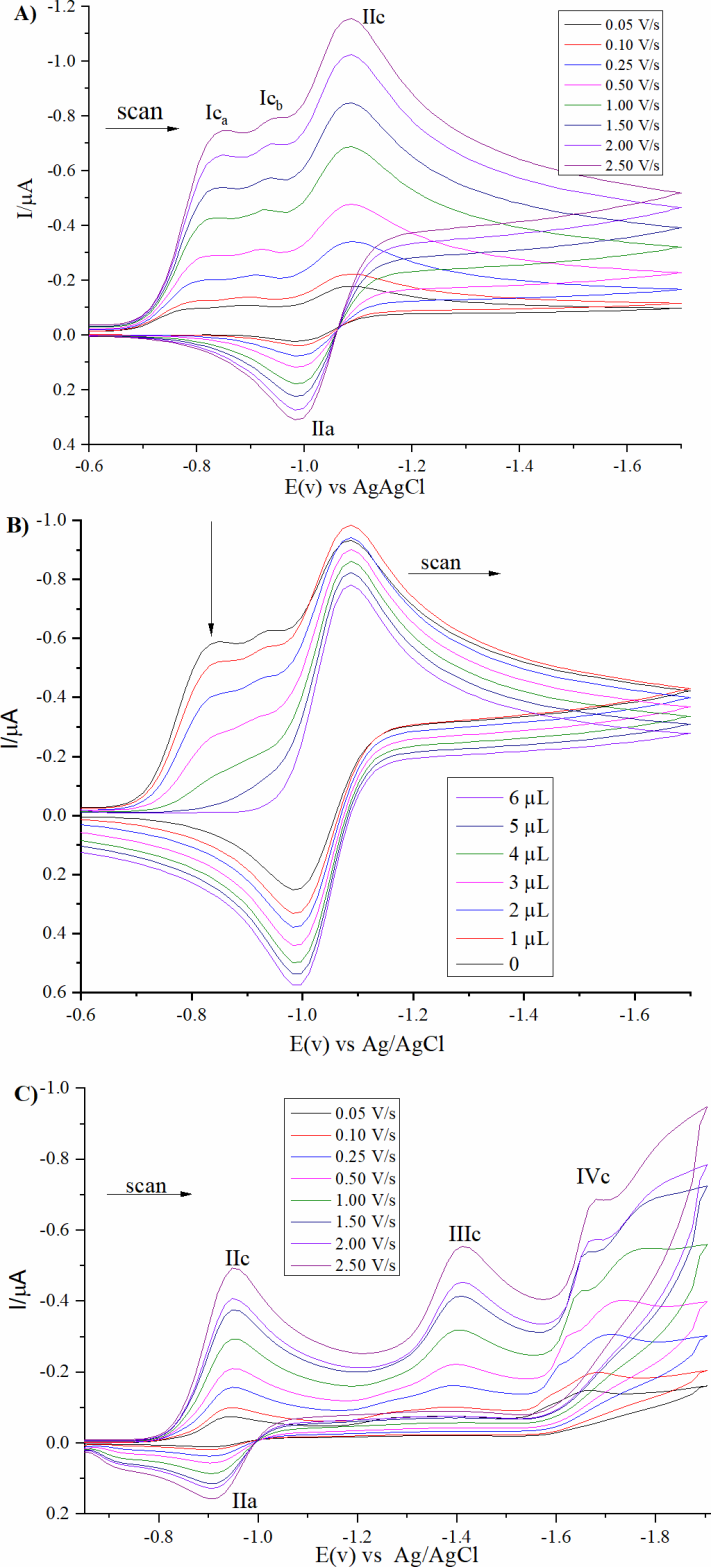
Cyclic voltammogram of (A) Compound 1 recorded at different scan rates between 0.05 and 2.50 V/s, (B) Compound 1 recorded at 2.00 V/s scan rate with the addition of different volumes of 0.1 M NaOH, and (C) Compound 5 recorded at different scan rates between 0.05 and 2.50 V/s. All the curves were recorded in DMSO as a solvent and PTBA as a supporting electrolyte.

**Fig 6:**
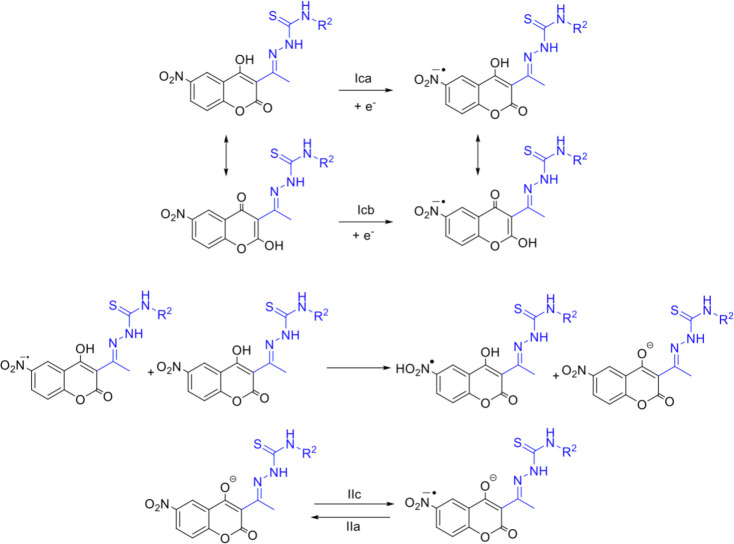
Proposed reduction mechanism for 6-nitrocoumarin-thiosemicarbazone hybrids 1–4.

Results indicated that compounds **1–4**, with a hydroxyl group in position 4, presented a similar electrochemical behavior. Figure 5A shows the cyclic voltammogram of compound **1** as a representative example. Three cathodic and one anodic signal were observed. The signals close to −0.88 and −0.99 V (Ic_a_ and Ic_b_, both irreversible) were attributed to the reduction of the nitro group of the two species involved in the keto-enolic equilibrium of the 4-OH coumarin moiety (48). The autoprotonation of the nitro group was evidenced by the addition of NaOH (Fig. 5B) (49). In fact, the third signal near −1.1 V (IIc, IIa, quasi-reversible) was attributed to the formation of the nitro anion radical. The proposed reduction mechanism is shown in Fig. 6.

**Fig 7:**
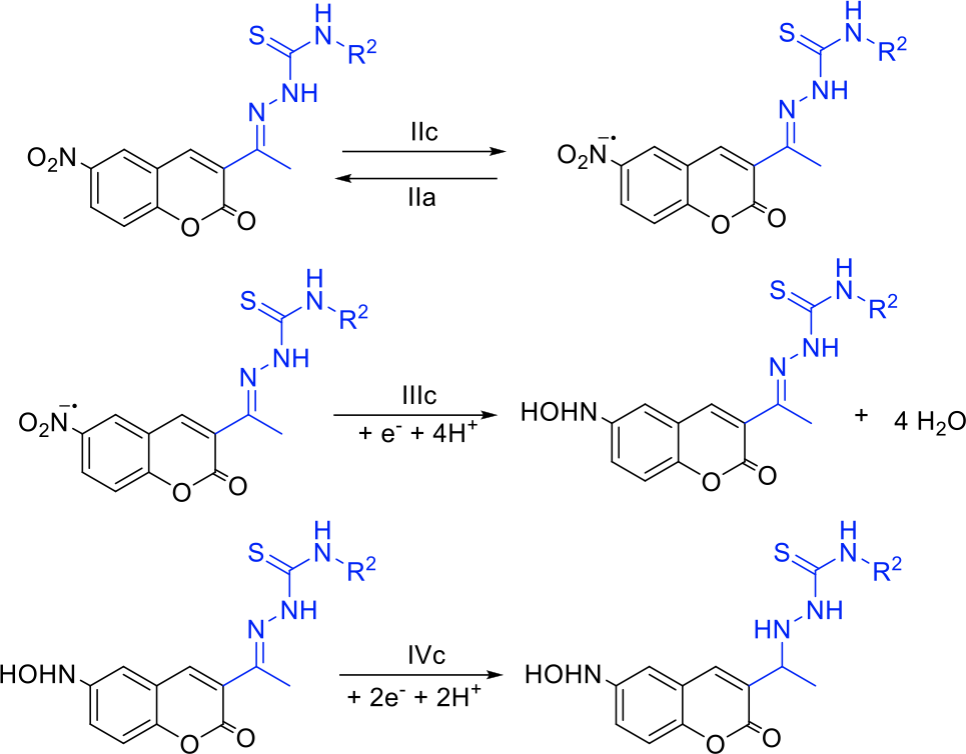
Proposed reduction mechanism for 6-nitrocoumarin-thiosemicarbazone hybrids 5–8.

Figure 5C exemplifies the electrochemical behavior of non-hydroxylated compounds **5–8**. Compound **5** has a quasi-reversible couple close to −0.94 V (IIa, IIc), which was attributable to the ${-NO}_{2}/{NO}_{2}^{.-}$ couple, the single-electron reduction of the nitro group. The irreversible signal close to −1.38 V (IIIc) was attributed to the formation of hydroxylamine and the irreversible signal close to −1.66 V (IVc) to the reduction via two electrons in the imine of the thiosemicarbazone moiety, similar to that described for 5-nitrofuryl containing thiosemicarbazones (50). Figure 7 shows the proposed reduction mechanism.

The reduction potentials of all the compounds (Table 1) are not affected by the substituent R^2^ in the thiosemicarbazone. Related to the reduction of the nitro moiety (couple II), the measured potential of the hybrid derivatives **1–8** was more negative than that of NFX, which could mean that the bioreduction could be less favorable. However, the measured potentials were similar to those reported for the 5-nitrofuryl thiosemicarbazone derivatives previously studied by the group (50, 51). For these compounds, the trypanocidal mechanism of action was associated with the generation of oxidative stress. Due to this, the prepared 6-nitrocoumarin-3-thiosemicarbazone hybrids **1–8** could be active against the infective form of *T. cruzi* through this mode of action.

**TABLE 1:** Redox potentials (vs. Ag/AgCl) corresponding to the reduction of hybrids 1–8, recorded at a rate of 2.00 V/s^*b*^

Compound	E(pIc_a_)	E(pIc_b_)	E(pIIc)	E(pIIIc)	E(pIVc)	E(pIIa)
1	−0.89	−0.99	−1.08	–^*c*^	–	−0.98
2	−0.89	−0.99	−1.08	–	–	−1.01
3	−0.88	−0.99	−1.08	–	–	−1.00
4	−0.62	−1.05	−1.09	–	–	−1.01
5	–	–	−0.94	−1.38	−1.66	−0.90
6	–	–	−0.93	−1.40	−1.68	−0.87
7	–	–	−0.94	−1.40	−1.67	−0.86
8	–	–	−0.94	−1.39	−1.68	−0.86
NFX^*a*^	–	–	−0.75	–	–	−0.70

^*a*^ Reference 52.

^*b*^ The potentials obtained in cyclic voltammetry were used to generate free radicals *in situ* electrolytically. ESR spectra were recorded to check the generation of the nitro anion radical for the obtained compounds. For all compounds, a nine-line hyperfine pattern was obtained (Fig. 8), whose hyperfine coupling constants (Table 2) were determined by computational simulation of the spectra using the WinSim program.

^*c*^ – indicates value not observed.

The comparison with the study of 3-nitrocoumarins reported by Salgado et al. shows key differences in the reduction potentials (32). While the 3-nitrocoumarins exhibited a more negative reduction potential (approximately −1.2 V), the 6-nitrocoumarins have less negative reduction potentials, suggesting a more straightforward reduction of these compounds than the 3-nitrocoumarins. This change in reduction potential is consistent with the structural modification at position 6 of the coumarin ring, which could facilitate the bioreduction of the nitro group in a biological environment and generate ROS with greater efficacy.

**Fig 8:**
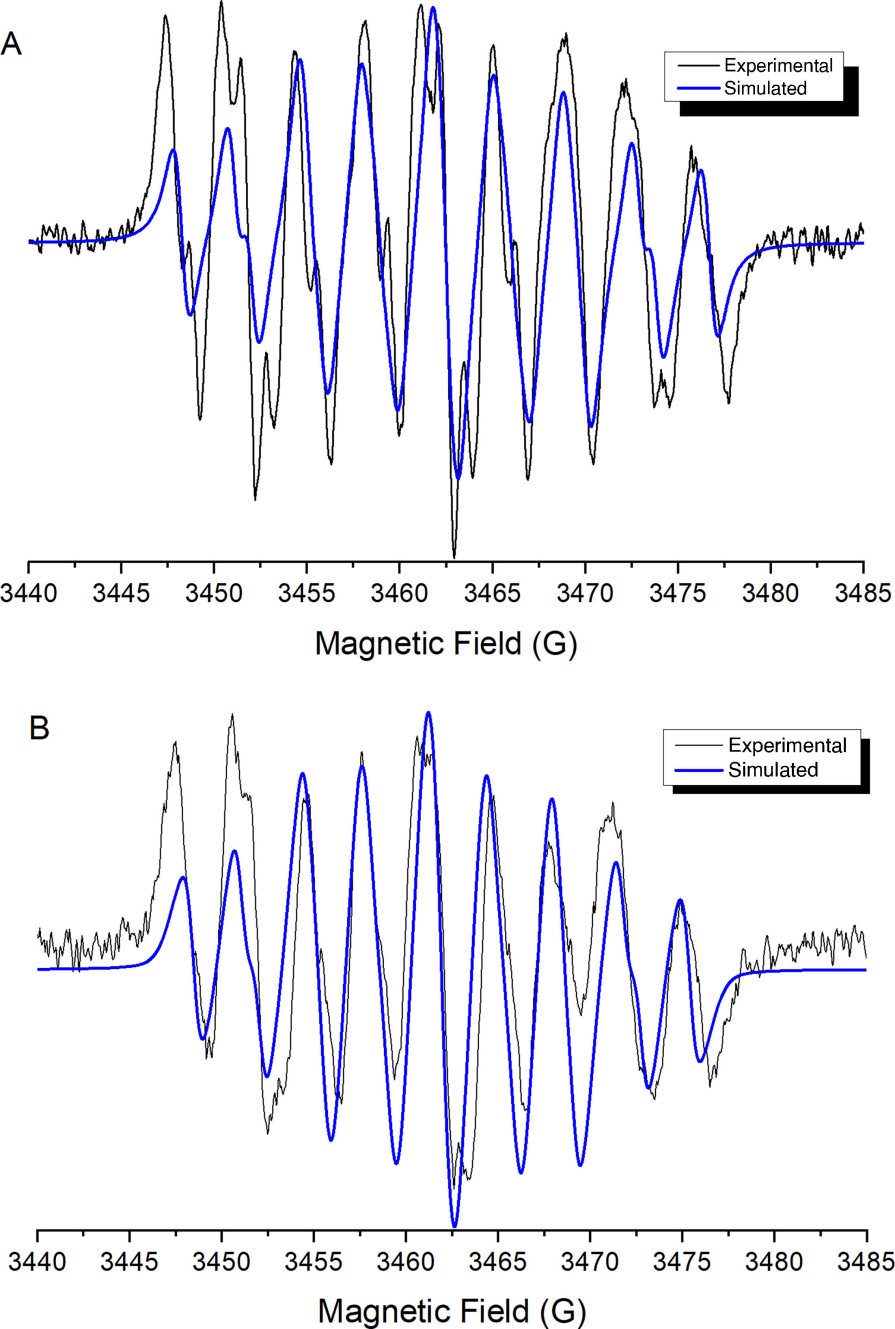
Experimental and semi-empirically simulated electron paramagnetic resonance spectra of (A) compound 1 and (B) compound 5.

**TABLE 2:** Hyperfine coupling constants of compounds 1–8 obtained by semi-empirical simulation

Compound	Hyperfine coupling constant (G)			
	a_N_	a_H-1_	a_H-2_	a_H-3_
1	7.08	7.54	3.87	3.09
2	7.08	7.61	3.80	2.86
3	7.09	7.56	3.81	2.96
4	7.07	7.74	4.11	3.19
5	6.59	6.87	4.09	3.22
6	6.87	7.21	4.07	2.97
7	6.78	7.21	3.62	2.66
8	6.98	7.36	4.01	2.49

The ESR spectra’s semi-empirical simulation considers the interaction between the nitrogen atom of the nitro group (aN = 7.05 G) and the three hydrogen atoms of the benzene ring in the coumarin backbone structure. In addition, in a previous study, we described how 3-nitrocoumarins (32), when reduced, present a less efficient delocalization of the unpaired electron due to the interference of the substituent at position 4, which hinders the efficient generation of ROS and leads to lower trypanocidal activity. By contrast, the compounds with the nitro group at position 6 show better stabilization of the generated radical, which could improve their biological activity.

The generation of these radical species partially confirms the proposed reduction mechanism for these compounds. These results suggest that the observed reduction potentials allow the bioreduction of these compounds, enabling oxidative stress as a potential mechanism of action. This hypothesis has been more extensively explored in the case of *Trypanosoma cruzi* (45).

### Biological evaluation

**Fig 9:**
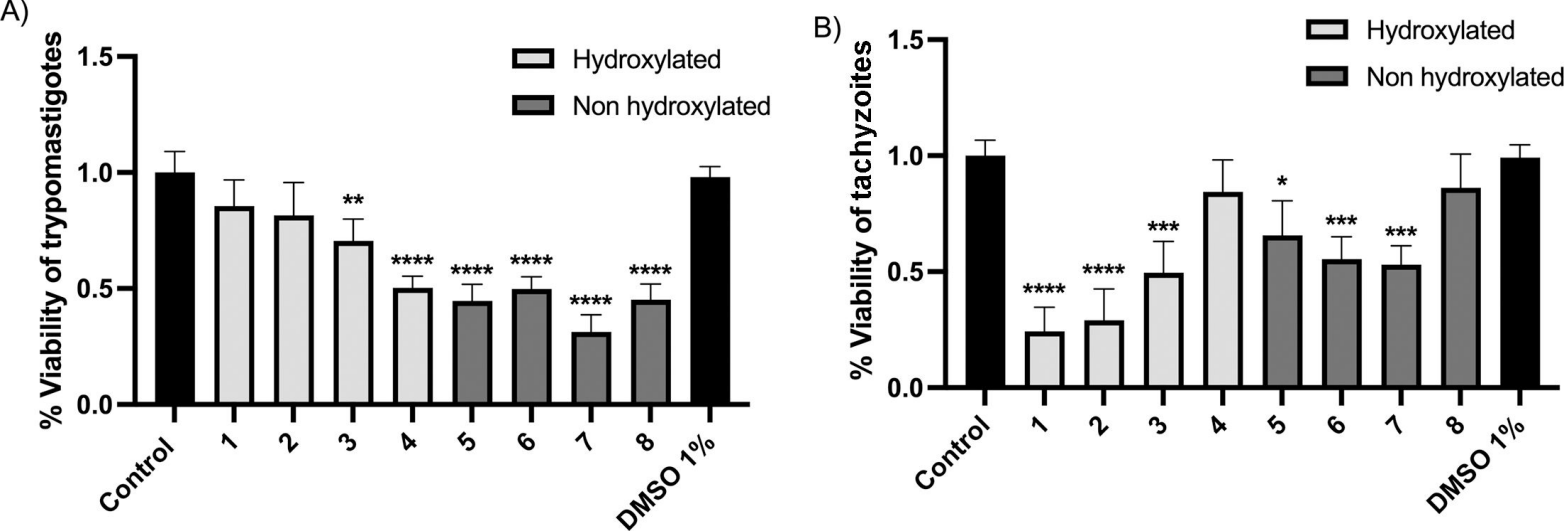
(A) Viability of *T. cruzi* trypomastigotes, Dm28 strain, exposed for 24 h to a concentration of 100 µM of compounds 1–8. (B) Viability of *T. gondii* tachyzoites, RH strain, exposed for 24 h to a concentration of 100 µM of compounds 1–8. The significant difference was compared to the negative uninfected control. One-way ANOVA with Dunnett’s post-test; ****P ≤ 0.0001; ***P ≤ 0.001; **P ≤ 0.01; *P ≤ 0.05.

The reduction of resazurin sodium salt was used to determine the trypanocidal and toxoplasmicidal activity of the 6-nitrocoumarin-thiosemicarbazone hybrids **1–8**. For this, treatment with the compounds was carried out for 24 h at a concentration of 100 µM. Most derivatives were, in general, more active against *T. gondii* than against *T. cruzi,* as shown in Fig. 9.

About the trypanocidal activity, the most active derivatives were those that do not contain a hydroxyl group in the 4 position of the coumarin (**5–8**), with compound **7** (which has an ethyl group as a substituent in the thiosemicarbazone moiety) being the most active. The hydroxylated compounds showed a marginal activity except for compound **4**, which is the most lipophilic of this series (Table 3). The relationship between lipophilicity and trypanocidal activity is unclear for the non-hydroxylated analogs, as the observed activity is quite similar for all of them, independently of the corresponding R_M_ values. The inclusion of the nitro moiety in the coumarin skeleton improves the anti-*T*. *cruzi* activity related to the previously reported non-nitrated derivatives (40, 41).

**TABLE 3:** Lipophilicity values (experimental RM and calculated log P) and antiparasitic activity on *T. cruzi* (trypomastigote) and *T. gondii* (tachyzoites) values at 100 µM for compounds **1–8**

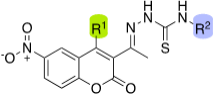						
Compound	R^1^	R^2^	Lipophilicity^*a*^ (R_M_)	Lipophilicity^*b*^ (log P)	Viability *T. cruzi* (%)	Viability *T. gondii* (%)
1	OH	H	0.00	1.44	85.5 ± 11.3	24.3 ± 10.4
2	OH	CH_3_	0.13	1.82	81.5 ± 14.1	29.1 ± 13.5
3	OH	CH_2_CH_3_	0.33	2.19	70.7 ± 9.4	49.6 ± 13.5
4	OH	Ph	0.85	2.88	50.3 ± 5.0	84.4 ± 13.7
5	H	H	−0.58	1.74	44.6 ± 7.1	65.7 ± 14.8
6	H	CH_3_	−0.41	2.11	49.8 ± 5.3	55.5 ± 9.6
7	H	CH_2_CH_3_	−0.39	2.49	31.3 ± 7.4	53.1 ± 8.1
8	H	Ph	0.20	3.17	45.2 ± 6.7	86.2 ± 14.4

^*a*^ TLC, reverse phase-C18, DMSO/phosphate buffer pH 7.4 (70:30 vol/vol) as mobile phase. R_M_ = log[(1/R_f_) – 1].

^*b*^ Obtained through the Molinspiration platform.

By contrast, our study evaluates the direct antiparasitic activity of new 6-nitrocoumarin-3-thiosemicarbazone hybrids against *T. cruzi* and *T. gondii* in cellular models and placental explants. The results demonstrate effective parasite elimination with a better selectivity profile, showing no significant toxicity in mammalian cells. This highlights the importance of testing compounds in biologically relevant models rather than relying solely on enzymatic assays, as potent enzyme inhibition does not always correlate with efficacy in parasite elimination. Our findings reinforce that while enzymatic inhibition is an important consideration, a more comprehensive evaluation, including cellular and tissue-based assays, is essential to determine the true therapeutic potential of new compounds (42).

The differences observed in the activities of both series of compounds (**1–4** vs. **5–8**) suggest that the inclusion of the hydroxyl group decreases the trypanocidal activity of this type of compound. This could be related to the potential antioxidant capacity of compound **1–4** since one of the potential trypanocidal mechanisms proposed for other nitro compounds (28), including NFX, is associated with the generation of oxidative stress in *T. cruzi*. Furthermore, a hydroxyl group in the structure would quench the radical species formed, reducing oxidative stress and trypanocidal activity.

Another possible explanation for the low activity displayed by hydroxylated derivatives could be the interaction with the parasite membrane. Compounds with a functional group capable of forming hydrogen bonds can establish a more significant interaction with the components of the cell membrane, preventing them from entering the parasite and efficiently acting on any intracellular target such as enzymes vital for survival or replication of the parasite (52–54). This interaction could compete with the increase in lipophilicity of the substituents linked to the thiosemicarbazone fraction, explaining the rise in activity for the most lipophilic compound of this series. Regarding non-hydroxylated compounds (**5–8**), a trend cannot be established between compound activity and their lipophilicity (Table 3) as the possible lipophilic interactions with the parasite cell membrane and, hence, the activity, could be modulated by the overall compound lipophilicity. Slight differences in lipophilicity caused by the substituents linked to the thiosemicarbazone fraction may not significantly impact compound activity.

On the other hand, a low relationship between trypanocidal activity and the IIc reduction potentials determined was observed. Despite this, a general trend was observed: the compounds with higher trypanocidal activity had lower reduction potentials. This could indicate a potential mechanism of trypanocidal action related to the generation of oxidative stress.

The toxoplasmicidal activity of all compounds on tachyzoites was also evaluated. Figure 8B shows that the most active compounds contain the hydroxyl group in position 4 of the basic structure of coumarin (**1–4**). In this parasite, the lipophilicity of the hydroxylated compounds seems to be indirectly proportional to the toxoplasmicidal activity against free tachyzoites, possibly related to differences in components of the parasite’s cell membrane between *T. cruzi* and *T. gondii* (55). For compounds **5–8**, as observed for *T. cruzi*, the changes in lipophilicity do not significantly affect the activity. However, it is evident that for both hydroxylated (**1–4**) and non-hydroxylated compounds (**5–8**), introducing a phenyl group in the structure of thiosemicarbazone worsened the toxoplasmicidal activity of the compounds.

Emami et al. described the toxoplasmicidal activity of a series of coumarin-quinolone derivatives (37), finding that compounds containing the hydroxylamine group exhibited high activity. However, in our study, we highlight that the thiosemicarbazone fraction plays a more crucial role in the biological activity of the derivatives, particularly in enhancing antiparasitic activity, compared to the coumarin portion, which was critical in the context of coumarin-quinolone derivatives. These results lead us to hypothesize that the thiosemicarbazone fraction plays a more significant role in the biological activity of these derivatives, potentially acting through a different mechanism, such as interference with the parasite’s metabolic pathways or inhibition of critical enzymes involved in parasite survival.

The effect of the compounds over intracellular amastigotes of a β-galactosidase-expressing recombinant strain of *T. cruzi* (Tulahuen β-gal) was also evaluated (see Table S4 at https://doi.org/10.5281/zenodo.15694236). Hydroxylated compounds (**1–4**) did not present activity over intracellular amastigotes, while the non-hydroxylated derivatives (**5–8**) showed an activity closely associated with their lipophilicity, with compound **8** being the most active. These results indicate that the compounds cross the mammalian cell’s plasma membrane and exert a trypanocidal effect over intracellular amastigotes. Similarly, intracellular anti-*T*. *gondii* tachyzoite was assessed using quantitative immunocytochemistry targeting the parasite’s P30 protein (see Table S4 at https://doi.org/10.5281/zenodo.15694236). Similar to what was observed regarding *T. cruzi*, hydroxylated compounds (**1–4**) exhibited no activity against intracellular parasites, likely due to their inability to traverse mammalian cell membranes. In this case, a modest activity against the intracellular forms of tachyzoites was observed for non-hydroxylated compounds (**5–8**) (see Table S4 at https://doi.org/10.5281/zenodo.15694236). The complete absence of activity of hydroxylated compounds against intracellular forms of both parasites was confirmed, which could be attributed to their inability to permeate mammalian cell membranes.

Furthermore, the pharmacokinetic properties of the compounds were evaluated using predictive computational tools from SwissADME (56), focusing on their potential oral bioavailability and their absorption, distribution, metabolism, and excretion (ADME) profile. The results, presented in Table S5 (see Table S5 at https://doi.org/10.5281/zenodo.15694236), indicate that the compounds have molecular weights ranging from 306.30 to 398.39 Da, with logP values between 0.55 and 2.55, within the range considered suitable for oral absorption according to Lipinski’s Rule (57). In addition, the number of hydrogen bond acceptors and donors met the criteria established by this rule (HBA ≤10 and HBD ≤5), suggesting a favorable profile for oral bioavailability (58).

Despite these favorable parameters, the topological polar surface area (TPSA) values ranged from 144.54 to 178.76 Å², exceeding the recommended limit of 140 Å². This could negatively affect intestinal permeability, as reported in previous studies on highly polar drugs (59). Solubility was evaluated using SwissADME models, which classified the compounds into soluble, moderately soluble, and poorly soluble categories. Compounds **1–7** were classified as soluble or moderately soluble, whereas compound **8** exhibited limited solubility, which could impact its oral absorption and *in vivo* bioavailability (60).

Metabolic interaction analysis indicated that compounds **4** and **8** inhibited the *CYP3A4* enzyme, suggesting a potential risk of pharmacokinetic interactions if co-administered with drugs metabolized by this cytochrome P450 isoform. None of the compounds showed significant inhibition of *CYP1A2, CYP2C19, CYP2C9,* or *CYP2D6*, indicating a lower likelihood of metabolic interactions with these other isoforms. Furthermore, according to the predictive model, the compounds exhibited low gastrointestinal absorption, likely due to their high TPSA values. In addition, none showed the ability to permeate the blood-brain barrier (BBB), suggesting that their action would be limited to the peripheral system, with no significant effects on the central nervous system (56). These limitations in predicted oral bioavailability and BBB penetration are relevant, particularly in the context of targeting *T. gondii* tissue cysts. However, these compounds are still under early-stage evaluation, and further structural optimization is underway. In addition, future studies will consider alternative delivery strategies, including nanoformulations and exosome-mediated transport, to enhance their pharmacokinetic behavior and tissue-targeting capabilities (61, 62).

From a structural perspective, compounds **5–8**, which lack hydroxyl groups, exhibited lower *TPSA* values and higher lipophilicity. This behavior correlated with greater trypanocidal activity in *in vitro* models, consistent with the hypothesis that greater permeability facilitates the compounds' access to intracellular parasites (60, 63). However, in *T. gondii*, the trend was different, as the most active compound **1** contained a hydroxyl group, suggesting differences in absorption and metabolism mechanisms between the two parasites.

On the other hand, compounds **4** and **8**, which inhibited CYP3A4 according to the ADME analysis, demonstrated notable activity, which could be linked to their ability to generate reactive oxygen species (ROS), a mechanism associated with their antiparasitic effect (64). The generation of ROS by nitro compounds against *T. cruzi* and *T. gondii* suggests that this mechanism may contribute to the observed efficacy (65, 66). Specifically, *T. gondii* has been shown to induce a state of oxidative stress in infected cells, supporting the hypothesis that ROS-generating compounds can effectively eliminate the parasite (66). Moreover, recent studies have confirmed that CYP3A4 inhibition can enhance ROS-mediated toxicity in parasitized cells, potentially explaining the differential activity observed in our assays.

By contrast, hydroxylated compounds (**1–4**) exhibited higher activity against *T. gondii*, which could be related to the presence of the hydroxyl group at position 4. This group stabilizes reactive species and reduces oxidative stress generation (67, 68). This suggests that, although oxidative stress may be a relevant mechanism of action, other factors, such as enzyme inhibition or alteration of essential metabolic processes in the parasite, may also contribute to its antiparasitic effect.

Finally, the physicochemical property profile of the evaluated compounds, presented in Fig. S1 (see Fig. S1 at https://doi.org/10.5281/zenodo.15694236), provides complementary information on their pharmacokinetic behavior and potential for future structural optimizations. These results suggest that additional modifications to the 6-nitrocoumarin-3-thiosemicarbazone hybrids could improve their pharmacokinetic profile and optimize their therapeutic efficacy against *T. cruzi* and *T. gondii*.

For the most active compounds, **7** and **1** (Table 3), the IC_50_ values against Dm28 *T. cruzi* trypomastigotes and RH strain *T. gondii* tachyzoites were determined. In addition, CC_50_ values were determined on human endothelial cells (EA.hy926) and the trophoblast cell line (BeWo). The last cell line was selected to obtain information regarding the potential application of the compounds in an *ex vivo* infection model (69). Results are shown in Table 4.

**TABLE 4:** IC_50_ values and selectivity indices for compounds **1** and **7** in human trophoblast cells (BeWo cells), human endothelial cells (EA.hy926), *T. cruzi* trypomastigote parasites of the Dm28 strain, and *T. gondii* tachyzoites of the RH strain

Compound	IC_50_ *T. cruzi *(μM)	IC_50_ *T. gondii* (μM)	CC_50_ EA.hy926 cells (μM)	CC_50_ BeWo cells (μM)	SI^*a*^	SI^*b*^
7	22.4 ± 0.8	–^*c*^	128.6 ± 0.7	277.9 ± 0.4	5.8	12.4
NFX	10 ± 0.4^*d*^	–	348.5 ± 1.2	>300	34.9	>30
1	–	17.3 ± 0.5	>400	386.9 ± 1.0	>23.1	22.3
PMN	–	20.8 ± 1.1	287.5 ± 0.9	125.5 ± 0.8	13.8	6.03

^*a*^ Selectivity index (SI) = EA.hy926 CC_50_ (μM)/parasite IC_50_ (μM).

^*b*^ Selectivity index (SI) = BeWo CC_50_ (μM)/parasite IC_50_ (μM).

^*c*^ – indicates value not determined.

^*d*^ Reference 70.

It should be noted that the IC₅₀ value reported for pyrimethamine in this table (20.8 ± 1.1 µM) was determined using a resazurin-based viability assay applied to extracellular *T. gondii* tachyzoites. This approach differs from the intracellular infection models commonly employed in the literature, which explains the discrepancy with the typically reported nanomolar values. The purpose of this measurement was solely to serve as an internal control under the same experimental conditions applied to the tested compounds. It should not be interpreted as a therapeutic benchmark.

Neither compound **7** nor compound **1** exhibited toxicity in mammalian cells, indicating good selectivity indexes. Regarding the BeWo cell line, the selectivity index for the *T. cruzi* activity obtained for compound **7** is comparable to the reference drug NFX. It is relevant to note that the inclusion of the nitro group in coumarin-thiosemicarbazone hybrids increased the anti*-T. cruzi* activity without increasing mammalian cell toxicity (40, 41). In the case of *T. gondii*, the results indicate that compound **1** has high activity against tachyzoites and low cytotoxic activity against BeWo cells, obtaining an SI like that obtained for PMN, considered a potential toxoplasmicidal compound. The CC_50_ curves of the compounds and drugs in the different mammalian cell lines are shown in Fig. S2 (see Fig. S2 at https://doi.org/10.5281/zenodo.15694236) and determined IC_50_ values on trypomastigotes and tachyzoites are shown in Fig. S3 (see Fig. S3 at https://doi.org/10.5281/zenodo.15694236).

These data were interpreted considering commonly accepted progression criteria in the antiparasitic drug discovery pipeline for *T. cruzi*, as discussed by Gabaldón-Figueira et al. (71). According to this framework, compounds with IC_50_ values below 5 µM (ideally <1 µM), selectivity index (SI) >10 (ideally >100), and cytotoxicity above 25 µM in mammalian cells are considered potential leads. While the IC₅₀ values of our compounds are in the micromolar range, they exhibit low toxicity in both endothelial and placental cells and satisfy selectivity thresholds, particularly in BeWo cells. Their ability to reduce parasite load in both 2D and *ex vivo* placental models supports their potential as chemical scaffolds for further optimization in the hit-to-lead phase.

It is essential to clarify that the primary objective of this work was not to present fully optimized drug candidates, but rather to explore a novel chemical scaffold with relevant biological activity, low cytotoxicity, and promising behavior in physiologically relevant models.

While the current potency is still moderate and further optimization will be required to improve efficacy and pharmacokinetic properties, the antiparasitic activity observed *in vitro*, along with the favorable toxicity profile, supports the value of these compounds as chemical starting points for future structure–activity relationship (SAR) studies.

The compounds evaluated in this study demonstrate significant antiparasitic activity against *T. cruzi* and *T. gondii*. This approach allows for a comprehensive assessment of their therapeutic potential, evaluating their effectiveness against different forms of parasites. The results highlight the broad-spectrum activity of the compounds, emphasizing their potential as effective therapeutic agents for the treatment of parasitic infections.

While the evaluated compounds demonstrated relevant activity in cellular models, identifying their mechanism of action is crucial for their optimization as potential therapeutic candidates. One possible mechanism to explain the observed activity is the generation of reactive oxygen species (ROS), as other coumarin derivatives have shown pro-oxidant effects in *T. cruzi* and *T. gondii*. To confirm this hypothesis, specific studies were conducted to quantify ROS formation in parasites treated with the most active compounds.

### Study of potential mechanisms of action

To evaluate a potential mechanism of action of the 6-nitrocoumarin-3-thiosemicarbazone hybrids, the formation of intracellular reactive oxygen species (ROS) was determined by quantifying the dichlorofluorescein (DCF) probe. ROS generation was evaluated in *Toxoplasma gondii* tachyzoites and *Trypanosoma cruzi* trypomastigotes, using the most active compounds for each parasite.

**Fig 10:**
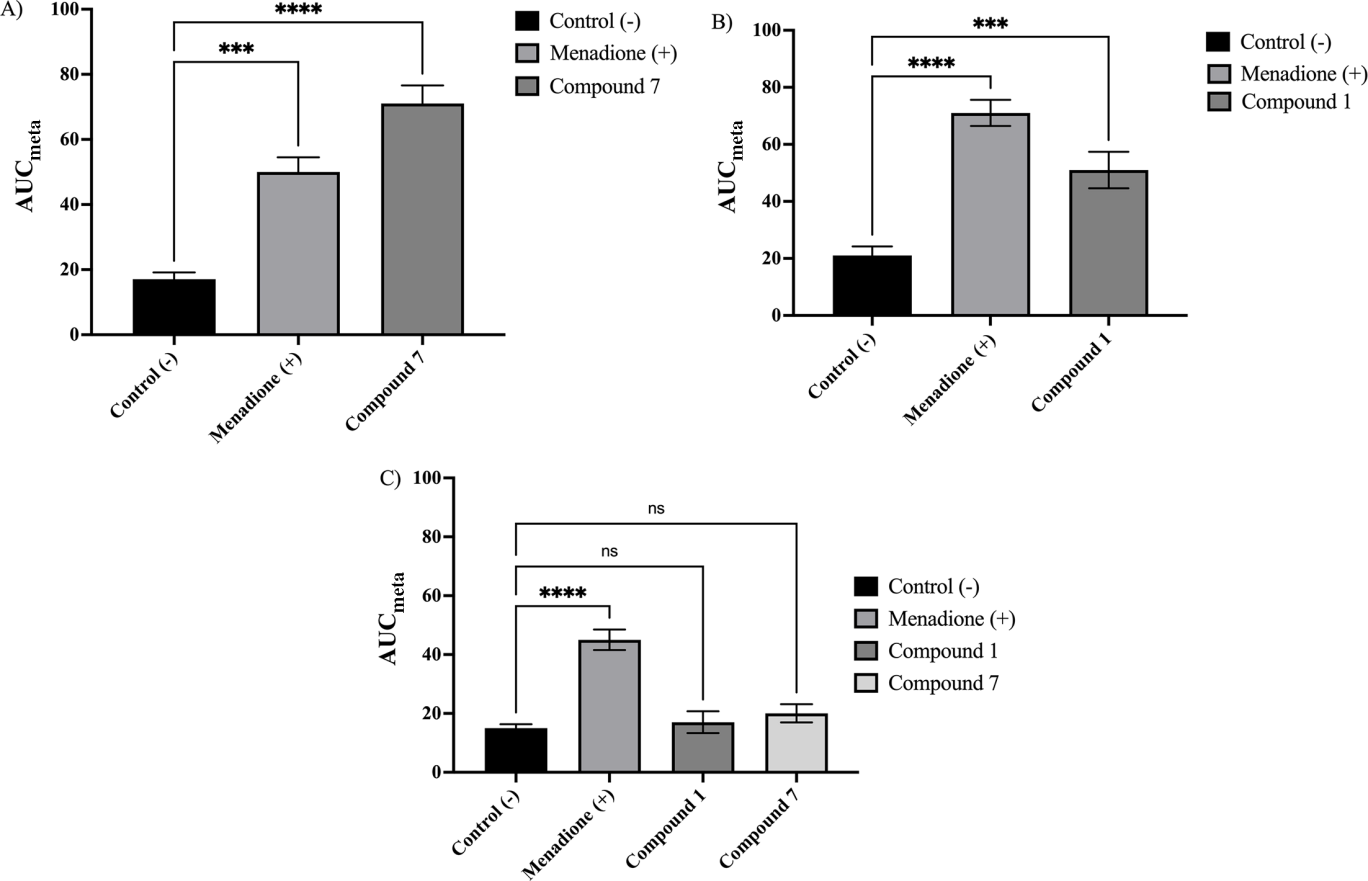
ROS were generated in *T. cruzi* and *T. gondii* after exposure to the evaluated compounds. (**A**) DCF fluorescence kinetics in *T. cruzi* trypomastigotes (strain Dm28) treated with compound 7, menadione (positive control), and untreated control. (**B**) DCF fluorescence kinetics in *T. gondii* tachyzoites (strain RH) treated with compound 1, menadione, and untreated control. (**C**) DCF fluorescence in BeWo cells after exposure to compounds 1 and 7, and menadione. The data represent the mean ± SD of three independent experiments (one-way ANOVA with Dunnett’s post hoc test; ****P ≤ 0.0001; ***P ≤ 0.001; **P ≤ 0.01; *P ≤ 0.05).

In the case of *T. cruzi*, compound **7** showed a significant increase in ROS generation compared to the untreated control, surpassing the effect observed for menadione (Fig. 10A). This result is consistent with previous studies that demonstrated the formation of intracellular radical species in coumarin-thiosemicarbazone hybrids without a nitro group (41). The incorporation of the nitro group appears to enhance oxidative stress generation, supporting its pro-oxidant mechanism of action in *T. cruzi* (32, 43, 45). In addition, electrochemical studies of these compounds revealed a more favorable reduction of the nitro group compared to nitro derivatives at position 3 of the coumarin. This could facilitate the formation of reactive species responsible for oxidative damage to the parasite (32).

In *T. gondii*, ROS formation was determined in RH strain tachyzoites using menadione as a positive control. A significant increase in ROS production was observed following exposure to menadione. At the same time, compound **1** also led to an increase in ROS levels, albeit lower than the positive control, but significantly higher than the untreated control (Fig. 10B). However, this compound contains a hydroxyl group at position 4 of the coumarin, which may be modulating its pro-oxidant effect. Hydroxyl groups have been described as antioxidants through the hydrogen atom transfer (HAT) mechanism, stabilizing reactive species and reducing oxidative stress generation (67, 68).

This behavior suggests that although oxidative stress may contribute to the antiparasitic activity of these compounds, it is not the sole mechanism involved in their action against *T. gondii*. The literature suggests that *T. gondii* relies on its antioxidant system to counteract oxidative damage induced by drugs (72). Since compound **1** induces lower ROS levels in *T. gondii* yet retains significant antiparasitic activity, other mechanisms, such as enzyme inhibition or interference with protein biosynthesis, may also be involved (72). This is consistent with previous studies on thiosemicarbazone compounds, where their activity has been proposed to be mediated by mitochondrial dysfunction and the inhibition of essential proteases required for parasite survival.

In addition, ROS generation was evaluated in BeWo cells to rule out non-selective cytotoxic effects (Fig. 10C). Unlike in parasites, the compounds did not induce a significant increase in ROS levels in these cells, suggesting a potential selectivity of action. This finding aligns with the low cytotoxicity observed in cell viability assays and supports the hypothesis that nitro group bioactivation occurs preferentially within the intracellular environment of the parasite.

These findings position the 6-nitrocoumarin-3-thiosemicarbazone hybrids as promising candidates for the treatment of *T. cruzi* and *T. gondii* infections, highlighting oxidative stress as a potential mechanism of action in *T. cruzi*. However, the lower ROS generation in *T. gondii* suggests that oxidative stress is not the exclusive mechanism, and other biological processes may contribute to its antiparasitic effect. The introduction of the nitro group into the coumarin core is a key strategy for enhancing trypanocidal activity. By contrast, the presence of the hydroxyl group at position 4 may modulate toxoplasmicidal activity by conferring antioxidant capacity.

Future studies should further evaluate the contribution of parasite antioxidant enzyme inhibition and potential interference with the redox machinery of *T. gondii* to validate whether oxidative stress is a complementary mechanism in this parasite or if these compounds act through a different pathway.

### Study of the generation of radical species by spin trapping

To characterize the reactive radical species formed and confirm oxidative stress as a possible mechanism of action, the spin-trap technique was used with DMPO in *Trypanosoma cruzi* trypomastigotes and *Toxoplasma gondii* tachyzoites, employing the most active compounds against each parasite.

**Fig 11:**
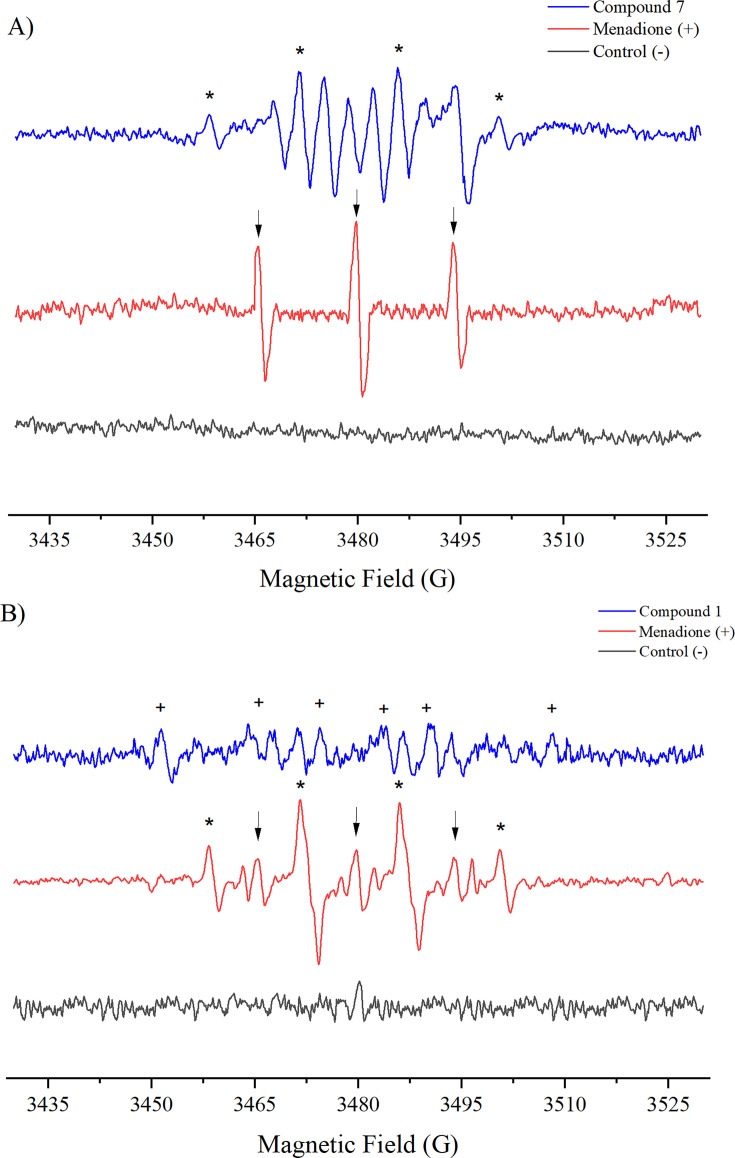
Electron spin resonance (ESR) spectra in *T. cruzi* and *T. gondii* after exposure to the tested compounds. (**A**) ESR spectrum recorded in *T. cruzi* trypomastigotes (strain Dm28) incubated with compound 7 and DMPO. Signals corresponding to hydroxyl radicals (+), carbon-centered radicals (*), and the oxidized adduct of DMPO (DMPOX; marked with ↓) are observed. (**B**) ESR spectrum recorded in *T. gondii* tachyzoites (strain RH) incubated with compound 1 and DMPO. Compared to *T. cruzi*, with carbon-centered radicals (*), DMPOX (↓), lower intensity signals are observed.

Figure 11A shows that incubation with menadione under aerobic conditions produced a triplet (aN ~15.5 G) corresponding to a DMPO-derived oxidized paramagnetic compound (DMPOX, 5,5-dimethyl-2-oxopyrroline-1-oxyl), marked with (↓) in the spectrum. This signal is related to the prior formation of hydroxyl radicals, as previously described in our studies (33, 73, 74).

Interestingly, treatment with compound **7** generated a series of complex signals associated with the formation of different free radicals. Among them, signals corresponding to hydroxyl radical generation (aN = aH ~ 14.00 G, marked with (*)) were identified (74, 75). In addition, signals attributable to the formation of the delocalized nitro radical in the aromatic system were detected, as had been recorded in the electrolytic generation of free radicals, and this was identifiable by hyperfine coupling constants in Table 2.

These signals suggest that compound **7** was reduced by the parasite’s nitroreductases, leading to the formation of radical species. This indicates a strong link between oxidative stress generation and the parasite’s antiparasitic mechanism of action. However, this mechanism does not rule out the possible involvement of other pathways, such as interference with key enzymes involved in parasite redox homeostasis.

The same experiment was conducted on *T. gondii* tachyzoites (Fig. 11B). Treatment with menadione (positive control) resulted in the formation of multiple signals, which were assigned based on their hyperfine coupling constants. A triplet (aN ~15.6 G) corresponding to a DMPO-derived oxidized paramagnetic compound (DMPOX) was detected, marked with (↓) in the spectrum. In addition, a sextet corresponding to the trapping of a carbon-centered radical (aN ~16.00 G, aH ~23.00 G, marked with (+)) was observed. A signal associated with hydroxyl radical generation (aN = aH ~ 14.00 G, marked with (*)) was also detected (33, 41, 45, 75).

On the other hand, treatment with compound **1** showed the formation of only two spin adducts, associated with the trapping of a carbon-centered radical and the DMPOX adduct. The lower number of radical species generated compared to *T. cruzi* reinforces the idea that while the toxoplasmicidal activity of these compounds may be related to oxidative stress generation, this may not be the sole mechanism involved.

The differences observed in the spectra of *T. cruzi* and *T. gondii* indicate variations in their endogenous antioxidant mechanisms (66, 76). It has been reported that *T. gondii* possesses a highly efficient redox system capable of neutralizing oxidative stress induced by pro-oxidant compounds (64). However, within the scope of this study, the formation of radical species in both parasites suggests that oxidative stress generation is a potential mechanism of action for these compounds.

Despite these findings, further studies are necessary to better understand the differences in each parasite’s response. Future research could focus on evaluating the expression of antioxidant genes in *T. gondii* and *T. cruzi* in response to these compounds and analyzing their enzymatic effect on parasite nitroreductases.

Based on the results obtained, it is postulated that the trypanocidal mechanism of 6-nitrocoumarin-3-thiosemicarbazone hybrids involves the bioreduction of the nitro group and the subsequent formation of free radicals in the parasite’s intracellular environment. A viable strategy for developing more effective and less cytotoxic trypanocidal and toxoplasmicidal drugs would be to retain the basic hybrid structure while optimizing its physicochemical properties through the formation of coordination complexes with transition metals, which could enhance its biological effect through additional bioactivation mechanisms.

### Effect of compounds on the infection of cells

The studies on ROS generation and electrochemical characterization suggest that the mechanism of action of these compounds may be related to the bioreduction of the nitro group and the induction of oxidative stress in *T. cruzi* and *T. gondii*. However, their impact on cellular and tissue infection was evaluated to confirm whether this effect directly contributes to parasite elimination in a biologically relevant environment. In addition, ADME analyses indicated that the lipophilicity of the compounds could influence their ability to cross cellular membranes and reach intracellular targets. Therefore, infection studies were conducted in trophoblast cells and placental explants to assess the efficacy of the compounds in reducing parasite burden, both in early infection stages and intracellular parasite forms.

The effect of the compounds on the infection of BeWo trophoblast cells by *T. gondii* and *T. cruzi* was assessed. The DNA of *T. gondii* and *T. cruzi* was quantified in infected BeWo cells treated for 24 hours under two conditions: co-incubation and post-infection treatment. The most active compounds, **1** and **7** for *T. gondii* and *T. cruzi*, respectively, were used at their IC_50_ concentrations, previously determined (Table 4). Fluorescence microscopy was employed to quantify the number of infected cells. The resulting images were analyzed using ImageJ software to count both infected and uninfected cells.

**Fig 12:**
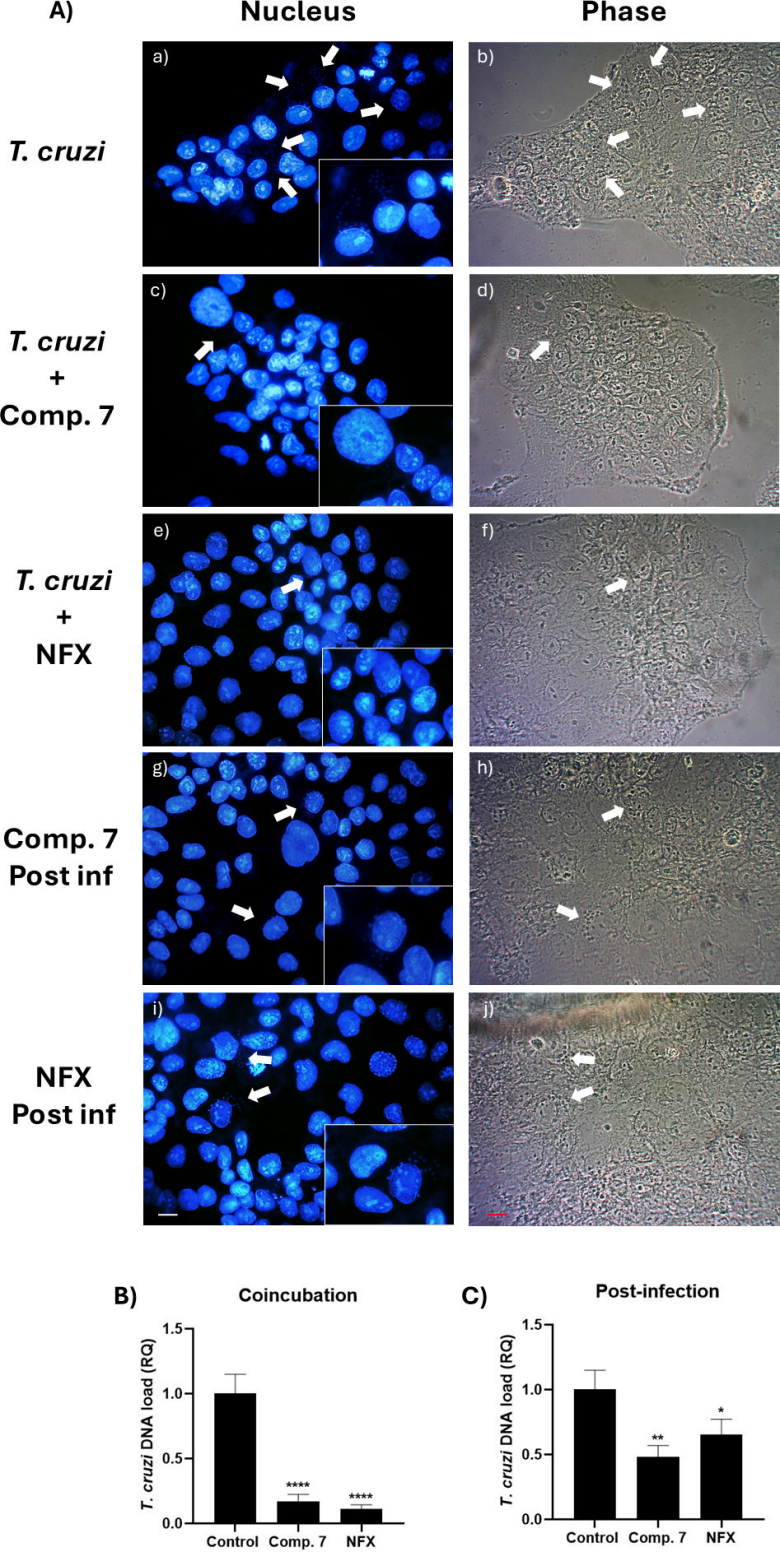
(A) Microscopic analysis of BeWo cells exposed to trypomastigotes of the Dm28 strain at a 1:1 ratio and treated with compound **7** and NFX at their IC_50_ concentrations. (a and b) Cells exposed to trypomastigotes without treatment, (c and d) co-incubation treatments with compound 7 or (e and f) NFX at the time of infection, (g and h) post-infection treatment with compound 7 or (i and j) NFX. Nuclear staining with DAPI for BeWo cell nuclei and *T. cruzi* kinetoplast (a, c, e, g, i), phase contrast (b, d, f, h, j); arrows indicate areas with amastigotes. The conditions of uninfected cells are shown found in Fig. S4 (see Fig. S4 at https://zenodo.org/records/15694236). Bar scale: 10 µm. (B) Relative quantification (RQ) of *T. cruzi* DNA load in BeWo after co-incubation with compound 7 and NFX at IC_50_ concentrations during a 24 hour infection. (C) Relative quantification (RQ) of *T. cruzi* DNA load in BeWo after 24 hour post-infection treatment with compound 7 and NFX at IC_50_ concentrations, following an initial 24 hour infection (one-way ANOVA with Dunnett’s post-test: (****P ≤ 0.0001; **P ≤ 0.01; *P ≤ 0.05).

Figure 12 shows the fluorescence microscopy images and parasite DNA levels by real-time qPCR of *T. cruzi-*infected trophoblast cells after different treatments. Co-incubation treatment with compound **7** reduced parasite DNA load by more than 50% compared to the negative control at IC_50_ concentration (Fig. 10B). Furthermore, the number of infected cells significantly decreased compared to the control without treatment (Table 5). These results are consistent with the activity observed against free parasites. The reduction in the infection rate of trophoblast cells during co-incubation suggests a potential inhibition of virulence factors involved in the parasitic invasion. However, we acknowledge that this observation might also reflect broader effects on parasite viability or energy metabolism. Since invasion is an active process that requires mitochondrial function and ATP availability, it is also possible that the observed inhibition results from impaired energetic processes. While our current data do not allow us to distinguish between these mechanisms, the significant reduction in infection still supports an early antiparasitic effect of compound **7**. Considering the previously documented activities of thiosemicarbazones, it is plausible that this effect is related to the inhibition of cruzipain, as described by Nunes for coumarin-3-thiosemicarbazone derivatives (42, 77, 78). Similar results had been obtained for the non-nitrated analogs (41).

**TABLE 5:** Determination of the effect of derivative 7 and NFX on the infection of BeWo cells by *T. cruzi* at the time of infection (co-incubation) and on the intracellular forms of the parasites (post-infection)^*a*^

Sample	Treatment	Infected cells (%)	Significance
Control	–^*b*^	24 ± 3	–
NFX	Co-inc	2 ± 1	****
7	Co-inc	4 ± 1	****
NFX	Post-inf	23 ± 4	NS
7	Post-inf	17 ± 4	*

^*a*^ Compound concentrations used correspond to the IC_50_ values previously obtained (one-way ANOVA with Dunnett's post-test; ****P ≤ 0.0001; *P ≤ 0.05; NS: non-significant).

^*b*^ – indicates not applicable (no treatment applied).

These results suggest that the observed decrease in parasite load could be related to the ability of the compounds to induce oxidative stress and affect parasite viability in the intracellular environment. Since these compounds have been shown to generate ROS, this effect may contribute to the inhibition of parasite proliferation in BeWo cells.

On the other hand, in the case of post-infection treatment (Fig. 12C), it was observed that compound **7** reduced the parasite DNA load to a lesser extent than during co-incubation, suggesting a lower activity against the intracellular form of *T. cruzi*. Table 5 shows that compound **7** slightly decreased the number of infected cells, as seen in Fig. 12. It is essential to highlight that these methodologies (DAPI nuclear staining and qPCR) allow quantifying the number of *T. cruzi* nuclei or parasite DNA, but do not discriminate between live or dead parasites. Therefore, these results indicate that compound **7** exhibited activity that decreased the intracellular proliferation of *T. cruzi* compared to the untreated control. The introduction of the nitro group in the coumarin fraction also improved the activity.

The expected activity was observed for NFX (Fig. 12), based on the fact that this drug usually has significant antiparasitic efficacy during the first weeks after infection, when the predominant form of the parasite is the trypomastigote. However, it shows reduced activity in posterior stages, where the intracellular form predominates. Compound **7** seems to behave similarly in treating both chronic and acute *T. cruzi* infections compared to NFX, with a tendency to perform better for the chronic one (7).

Compound concentrations used correspond to the IC_50_ values previously obtained (one-way ANOVA with Dunnett’s post-test; ****P ≤ 0.0001; *P ≤ 0.05; NS: non-significant).

**Fig 13:**
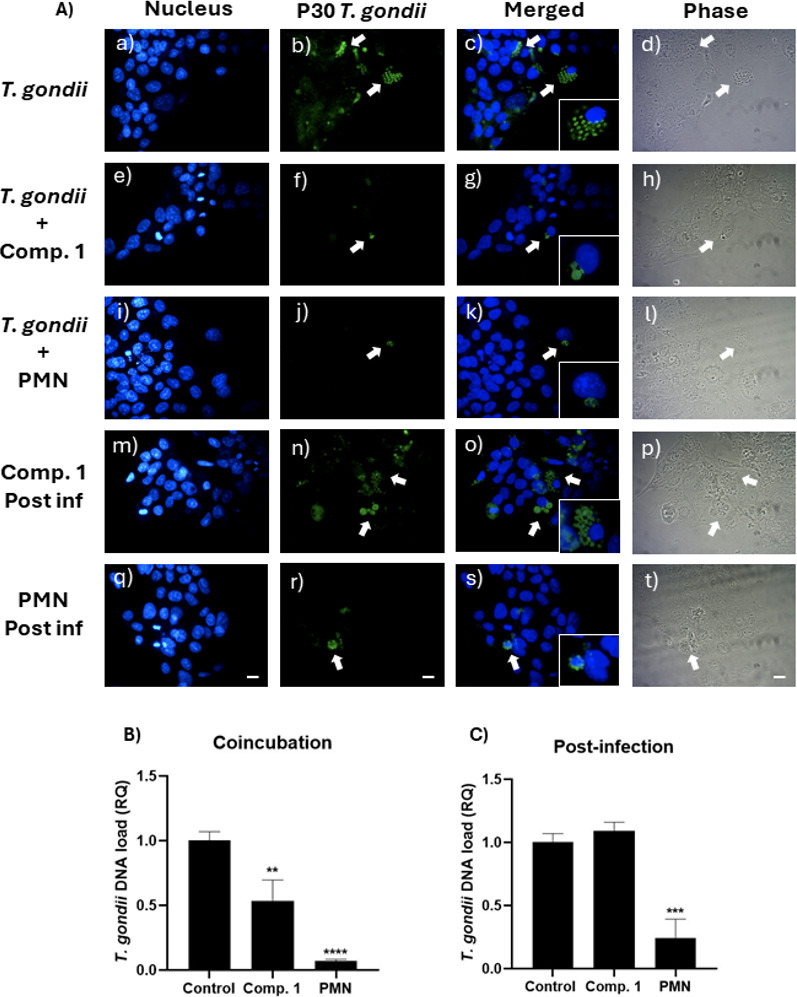
(A) Microscopic analysis of BeWo cells exposed to tachyzoites of the RH strain at a 1:1 ratio and treated with compound 1 and PMN at their IC_50_ concentrations. (a–d) cells exposed to tachyzoites without treatment, (e–h) co-incubation treatments included compound 1 or (i–l) PMN at the time of infection, (m–p) post-infection treatments were applied 24 hours later with compound 1 or (q–t) PMN. Nuclear staining with DAPI for BeWo cell nuclei (a, e, i, m, and q). Immunofluorescence of P30 protein of *T. gondii* (b, f, j, and r). Merged DAPI and immunofluorescence (c, g, k, o, and s). Phase contrast (d, h, l, p, and r). Arrows indicate areas of major immunoreactivity. The conditions of uninfected cells are shown in Fig. S5 (see Fig. S5 at https://zenodo.org/records/15694236). The conditions of uninfected cells and cells not treated with anti-P30 antibody are shown in Fig. S5 (see Fig. S5 at https://zenodo.org/records/15694236). Bar scale: 10 µm. (B) Relative quantification (RQ) of *T. gondii* DNA load in BeWo after co-incubation with compound 1 and PMN at IC_50_ concentrations during a 24 hour infection. (C) RQ of *T. gondii* DNA load in BeWo after a 24 hour post-infection treatment with compound 1 and PMN at IC_50_ concentrations, following an initial 24 hour infection (one-way ANOVA with Dunnett’s post-test: (****P ≤ 0.0001; ***P ≤ 0.001; **P ≤ 0.01).

For the infection of trophoblast cells, the treatment for *T. gondii* infection in co-incubation with compounds **1** and PMN reduced the parasite DNA load (Fig. 13A B). A decrease of around 50% in the parasite DNA load in co-incubation with compound **1** was observed, while the effect of PMN was more significant. These results are consistent with those obtained for the evaluation of toxoplasmicidal compounds using the free parasite and a sensitive redox agent such as resazurin. Likewise, co-incubation with compound **1** also reduced the rate of infection of BeWo cells (Table 6). It did not reduce the immunoreactivity for *T. gondii* in the infected cells, which initially demonstrates a disadvantage of this compound against PMN.

**TABLE 6:** Determination of the effect of compound 1 and PMN on the infection of BeWo cells by *T. gondii* at the time of infection (co-incubation) and the intracellular forms of the parasites (post-infection)^*a*^

Sample	Treatment	Infected cells (%)	Significance
Control	–^*b*^	64 ± 6	–
PMN	Co-inc	19 ± 2	****
1	Co-inc	32 ± 7	****
PMN	Post-inf	16 ± 4	****
1	Post-inf	52 ± 4	*

^*a*^ Compound concentrations used correspond to the IC_50_ values previously obtained (one-way ANOVA with Dunnett's post-test; ****P ≤ 0.0001; *P ≤ 0.05; NS: not significant).

^*b*^ – indicates not applicable (no treatment applied).

In the case of post-infection treatment, PMN significantly reduced the parasite DNA load of *T. gondii* (Fig. 13C). However, compound **1** had no activity against the intracellular form.

Regarding the post-infection treatment, the results obtained through the quantification of intracellular parasites using fluorescence microscopy (Fig. 13A) show a statistical similarity with the values obtained in the quantification by parasite DNA loading in the *in vitro* model, where it is confirmed that compound **1** does not present a toxoplasmicidal activity by reducing infected cells, which is satisfactorily explained by the inability of hydroxylated compounds of permeating the mammalian cell membrane. Hence, they did not have intracellular activity. Despite this, the result highlights the importance of considering this physicochemical characteristic for the rational design of toxoplasmicidal compounds or the possibility of improving bioavailability using drug vehicles that can facilitate the entry of this type of compound into the infected cell, potentially allowing for direct action against *T. gondii*.

In addition, the limited effect observed for compound **1** in post-infection treatment may reflect a restricted activity window associated with specific stages of the *T. gondii* lytic cycle. Recent studies have demonstrated that thiosemicarbazones can induce a transient, non-genetic adaptation in *T. gondii*, characterized by stress-induced changes in the expression of transporters and ribosomal proteins (62). Although our data do not support stable resistance, it is possible that compound 1 acts primarily during invasion or early intracellular processes, rather than during sustained replication. Future optimization efforts will focus on structural modifications designed to improve intracellular efficacy and evaluate potential adaptive responses more specifically.

Our data show compounds **5–8** were active against intracellular stages of two *T. cruzi* strains belonging to distant genetic lineages: Dm28 (TcI) and Tulahuen (TcVI). It is tempting to speculate that the studied compounds would also be active against other, genetically different, strains of *T. cruzi*. Experimental confirmation of this claim warrants further studies.

On the other hand, the lower activity observed in the post-infection condition could be related to the difficulty of the compounds crossing cell membranes and reaching their intracellular targets. Furthermore, in *T. cruzi* and *T. gondii*, the presence of antioxidant systems could contribute to the intracellular forms’ resistance to oxidative stress induced by the compounds.

### Effect of compounds in the infection of HPE

The infection of HPE by *T. cruzi* and *T. gondii* has revealed differentiated immune responses and defense mechanisms, highlighting the complexity of the pathogen-host interaction in this tissue (79). Among the primary defense mechanisms observed for both pathogens is the detachment of the trophoblast from the free chorionic villi. However, this mechanism may not stop the progress of the infection, resulting in significant tissue damage (80, 81). This damage is characterized by the disorganization of the extracellular matrix, evidenced by the loss of integrity in the distribution of key extracellular matrix components such as collagen and fibronectin. Thus, the HPE model has been widely used to study pathogen-host interactions and to evaluate drugs and compounds with antiparasitic activity, especially in the case of *T. gondii* (8). This model offers an advantage over traditional *in vitro* models by allowing detailed histopathological analysis, providing a more comprehensive view of the effects of drugs in terms of both efficacy and toxicity.

The effect on HPE infection of compounds **1** and **7** by *Toxoplasma gondii* and *Trypanosoma cruzi*, respectively, was evaluated. To do this, the DNA of the *T. gondii* or *T. cruzi* parasite was quantified by infecting HPE for 24 hours under treatment conditions of co-incubation of compounds with the parasite at the same time of infection, and with a 24 hour post-infection treatment. The compound’s IC_50_ concentration value previously obtained under both conditions was used.

**Fig 14:**
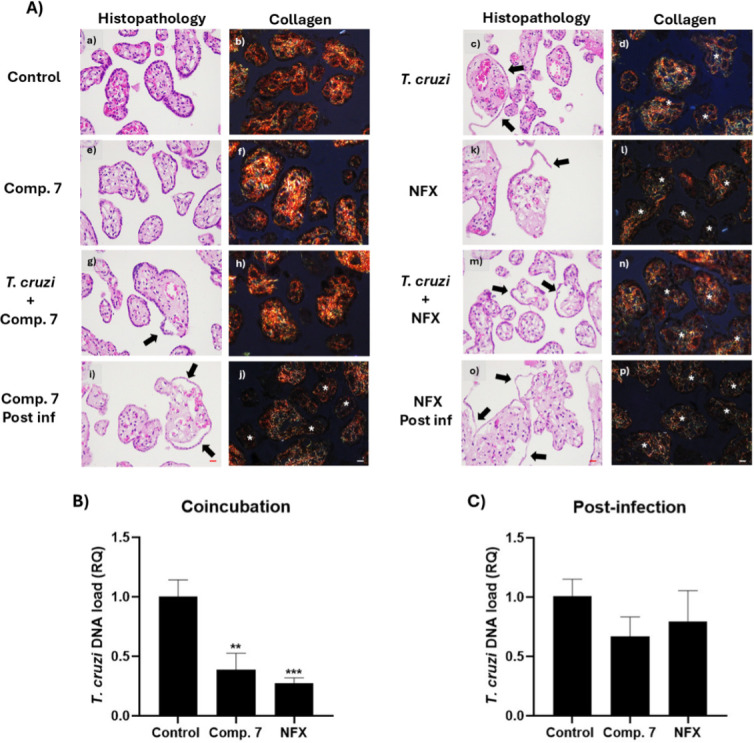
(A) Histological analysis of HPE exposed to *T. cruzi*, compound 7, and NFX at their respective IC_50_ values. HPE were incubated either in the absence (a and b, e and f, k and l) or presence (c and d, g–j, m–p) of 10^5^ trypomastigotes/mL of the Dm28 strain for 24 hours. Co-incubation treatments included compound 7 (g and h) or NFX (m and n) at the time of infection. Post-infection treatments were applied 24 hours later with compound 7 (i and j) or NFX (o and p). HPE, in the absence of trypomastigotes, was exposed to compound 7 (e and f) or NFX (k and l) for 24 h to observe the direct effect of the compounds on the HPE. Tissues were processed for routine hematoxylin-eosin histology (a, c, e, g, i, k, m, and o) and Picro Sirius red collagen histochemistry (b, d, f, h, j, l, n, and p). Arrows indicate the areas where the trophoblast is detached, and asterisks indicate the areas where birefringence reduction is present in PRS-stained samples. Bar scale: 20 µm. (B) Relative quantification (RQ) of *T. cruzi* DNA load in HPE after co-incubation with compound 7 and NFX at IC_50_ concentrations during a 24 hour infection. (C) Relative quantification (RQ) of *T. cruzi* DNA load in HPE after a 24 hour post-infection treatment with compound 7 and NFX at IC_50_ concentrations, following an initial 24 hour infection (one-way ANOVA with Dunnett’s post-test: (***P ≤ 0.001; **P ≤ 0.01).

Figure 14 shows the effects of compound **7** on the DNA load of the *T. cruzi* parasite in co-incubation and post-infection treatments, respectively, as well as the histological images of the HPE with the different treatments. The co-incubation treatment significantly reduced the parasite DNA load, showing a trend like that observed in the *in vitro* model. However, in this model, a higher parasite load was recorded for co-incubation compared to what was observed in BeWo cells, suggesting that the parasite, when coincubated with the compounds, could have a greater entry capacity to tissue compared to its ability to infect BeWo cells.

Regarding post-infection treatment, no statistically significant activity against the intracellular form was detected neither for compound **7** nor NFX, despite showing a trend that could represent an advantage of compound **7** over NFX. This could be explained because, in this model, the compound must permeate not only the membranes of the host cells but also more complex structures such as the extracellular matrix, which presents collagen, elastic fibers, proteoglycans, and glycoproteins, among others. However, the expected difference in activity between co-incubation and post-infection was observed for NFX, as this drug has weak activity against the intracellular form, similar to what was observed in the *in vitro* model. For compound **7**, results were also similar to those recorded in the *in vitro* model.

A histopathological study was conducted on the free chorionic villi of HPE under the treatment conditions described in Fig. 14. The co-incubation treatment with compound **7** prevented tissue damage induced by trypomastigotes, avoiding trophoblast detachment (indicated by arrows) and maintaining the structural integrity and collagen detection in the extracellular matrix. In comparison, compound **7** showed advantages over the reference drug NFX. Although NFX, under co-incubation conditions, managed to reduce the parasitic load, it also caused tissue damage under the treatment conditions, and even when explants were incubated only with the drug. By contrast, compound **7** did not induce tissue damage under these conditions. However, despite compound **7** showing a slight reduction in the parasitic load in the post-infection treatment conditions, possibly attributable to an intracellular effect of the compound, no reduction in tissue damage caused by trypomastigotes was observed in this condition, with a decrease in collagen indicating a reduction in the integrity of the extracellular matrix.

About the above, Masson’s trichrome staining (see Fig. S6 at https://doi.org/10.5281/zenodo.15694236) and Picro Sirius Red staining revealed damage to the extracellular matrix. The reduction in birefringence observed with the latter indicates disorganization of type I collagen, the main component of the extracellular matrix. Although compound **7** exhibits an anti-*T*. *cruzi* effect, as evidenced by the reduction of parasitic DNA load in the *in vitro* model, tissue damage persists. This damage is associated with the invasion process, suggesting that post-infection treatment does not prevent it. However, the results also show that the integrity of the extracellular matrix is preserved with the co-incubation treatment. This could be explained either by the parasite’s death before it invades the tissue or by the inhibition of invasion, possibly due to the suppression of the parasite’s virulence factors. These findings suggest that compound **7** could serve as a scaffold for designing more potent trypanocidal agents, which could be useful in the treatment of Chagas disease.

**Fig 15:**
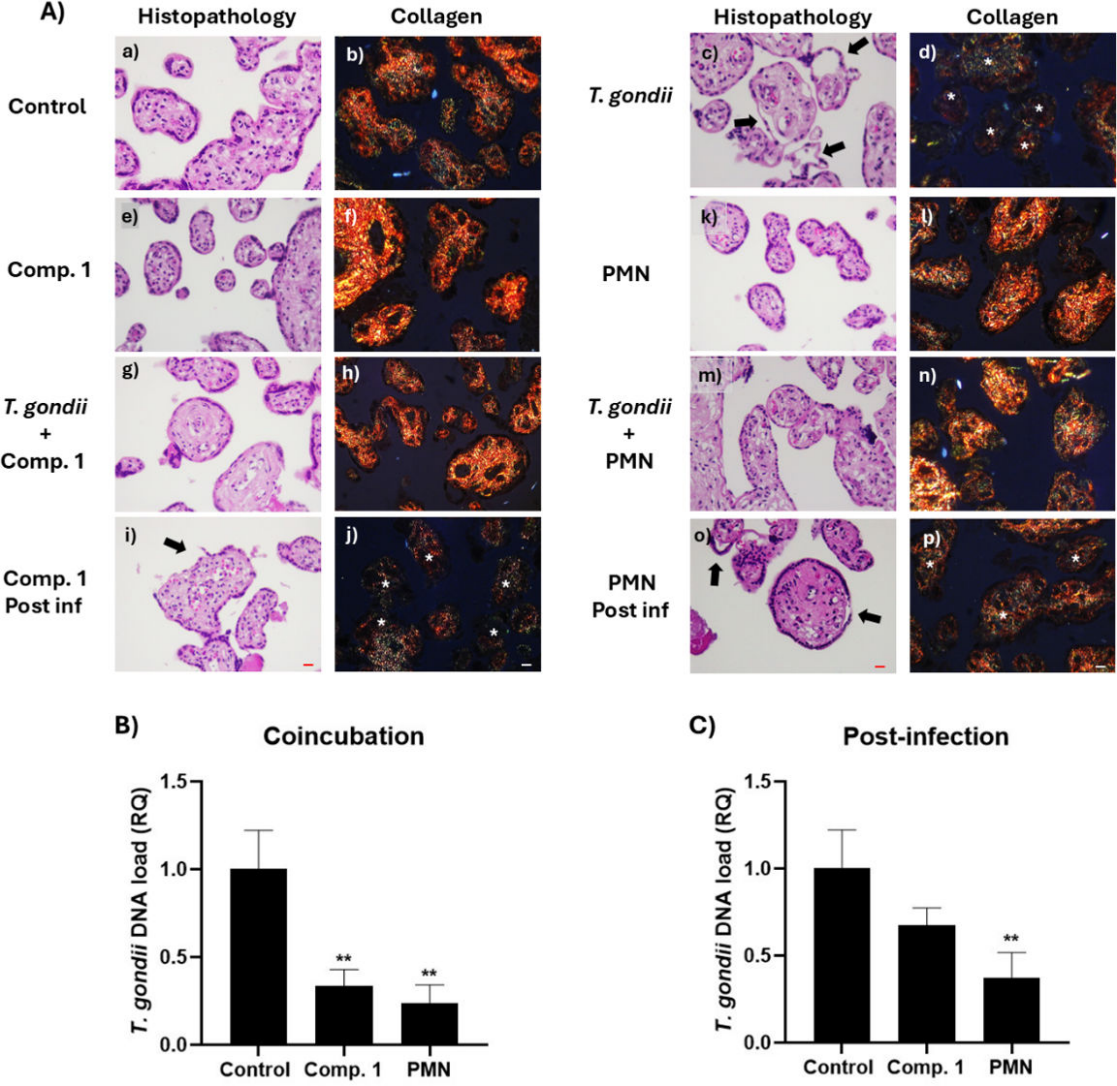
(A) Histological analysis of HPE exposed to *T. gondii*, compound 1, and PMN at their respective IC_50_ values. HPE were incubated either in the absence (a and b, e and f, k and l) or presence (c and d, g–j, m–p) of 10^5^ tachyzoites/mL of RH strain for 24 hours. Co-incubation treatments included compound 1 (g and h) or NFX (m and n) at the time of infection. Post-infection treatments were applied 24 hours later with compound 1 (i and j) or PMN (o and p). HPE, in the absence of tachyzoites, was exposed to Comp 1 (e and f) or PMN (k and l) for 24 h to observe the direct effect of the compounds on the HPE. Tissues were processed for routine hematoxylin-eosin histology (a, c, e, i, j, k, m, o) and Picro Sirius red collagen histochemistry (b, d, f, h, j, l, n, p). Arrows indicate the areas where the trophoblast is detached, and asterisks indicate the areas where birefringence reduction is present in PRS-stained samples. Bar scale: 20 µm. (B) Relative quantification (RQ) of *T. gondii* DNA load in HPE after co-incubation with compound one and PMN at IC_50_ concentrations during a 24 hour infection. (C) Relative quantification (RQ) of *T. gondii* DNA load in HPE after a 24 hour post-infection treatment with compound one and PMN at IC_50_ concentrations, following an initial 24 hour infection (one-way ANOVA with Dunnett’s post-test; **P ≤ 0.01).

Figure 15B C shows the effects of compound **1** on the DNA load of the *T. gondii* parasite in co-incubation and post-infection treatments at IC_50_ concentration, maintaining a similar trend to that observed in the *in vitro* model. For the 24 hour post-infection treatment, the parasitic DNA load decreased for the PMN treatment and, to a lesser extent, for the derivative **1** treatment, which is not statistically significant, as occurred in the *in vitro* infection model.

Histopathological analysis was conducted on the free chorionic villi of HPE under the treatment conditions specified in Fig. 15A. Co-incubation treatment with compound **1** prevented tissue damage induced by tachyzoites, avoiding trophoblast detachment (indicated by arrows) and preserving structural integrity and collagen detection in the extracellular matrix according to the observations made by Masson’s trichrome (see Fig. S6 at https://doi.org/10.5281/zenodo.15694236) and Picro Sirius red staining. In comparison, compound **1** did not demonstrate superiority over the reference drug PMN, effectively reducing the parasitic load under the same co-incubation conditions without causing tissue damage—neither compound induced tissue damage when incubated with the explants, confirming the low toxicity of compound **1**.

In the context of post-infection treatment, it was observed that compound **1**, like the *in vitro* model, did not reduce the parasitic load or mitigate tissue damage caused by the parasite. This was reflected in a decrease in the integrity of the extracellular matrix, evidenced by reduced collagen levels and increased trophoblast detachment in the explants. Conversely, post-infection treatment with PMN effectively reduced the explants’ parasitic load and decreased the induced tissue damage. This effect is attributed to PMN, which seems to inhibit the invasion process even 24 hours after infection, resulting in reduced tissue damage and DNA load compared to the untreated control. This phenomenon could explain the slight decrease in DNA load observed in the post-infection treatment with compound **1**, although not statistically significant, in contrast to the results obtained in the *in vitro* model.

The results obtained in placental explants suggest that oxidative stress may be involved in the antiparasitic effect of these compounds. The reduction in parasite burden observed under co-incubation conditions indicates that the generation of ROS could contribute to parasite inhibition before tissue invasion (72, 76). However, in the post-infection condition, the lower efficacy of the compounds might be related to limitations in their ability to cross the cell membrane and extracellular matrix of placental tissue. In addition, *T. cruzi* and *T. gondii* possess antioxidant systems that could mitigate the impact of oxidative stress induced by these compounds, potentially explaining the reduced activity observed against intracellular parasite forms. These findings highlight the need for further investigation into the relationship between oxidative stress, tissue permeability, and parasite resistance in this model.

Our studies on evaluating potential new drugs demonstrate that the *ex vivo* model is a useful tool for the preliminary assessment of tissue damage that compounds may cause. Furthermore, the results show a similar trend in the effects on infection in trophoblast cells and human placental explants, suggesting that the *in vitro* model could effectively predict parasitic load in more complex tissues, such as the placenta. However, utilizing a more accessible and less complex *in vitro* model than human placental explants could be valuable for a broader analysis of compound families. In this regard, 3D models represent an interesting alternative to explore for obtaining an *in vitro* model that accurately reflects placental tissue.

In this context, although the results obtained in the placental explant model confirm the trends observed in the *in vitro* assays, this consistency should not be viewed as methodological redundancy, but rather as a physiological validation of the simpler model. This coherence enhances the value of the *in vitro* system as an effective initial filter for evaluating antiparasitic compounds. At the same time, the explant model offers the opportunity for more in-depth investigations, including parasite-specific immunolocalization and detailed phenotypic characterization of tissue interactions. This perspective has been incorporated into the study’s future outlook, focusing on the development and validation of optimized compounds.

Furthermore, future efforts will include pharmacokinetic and metabolic studies to evaluate compound stability, bioavailability, and systemic exposure. These analyses will be crucial in determining whether therapeutic concentrations can be achieved *in vivo*. In addition, resistance profiling will be crucial in assessing potential parasite adaptation under prolonged exposure, particularly for structurally optimized derivatives with enhanced potency.

Although *in vivo* evaluation remains a critical step in antiparasitic drug development, we considered that at this early stage, with limited potency and no pharmacokinetic data, such studies would not yield conclusive results. Therefore, future *in vivo* studies will be reserved for structurally optimized compounds with improved biological profiles and predicted drug-like properties, to ensure relevance and translatability of the findings.

### Conclusion

Eight new 6-nitrocoumarin-3-thiosemicarbazone hybrids were synthesized and characterized, evaluating their activity against *Trypanosoma cruzi* and *Toxoplasma gondii* in cellular and tissue models. The most active compounds, **1** and **7**, significantly reduced parasite burden, especially under co-incubation conditions. However, their post-infection effectiveness was lower, possibly due to limitations in cellular penetration or resistance mechanisms of the parasites.

The compounds exhibited low cytotoxicity in mammalian cells and did not cause tissue damage in placental explants, indicating a favorable selectivity profile. Electrochemical and ESR studies suggest that bioreduction of the nitro group and generation of ROS may play roles in their mechanism of action against *T. cruzi*. Additionally, other processes, such as enzymatic inhibition, may be involved in combating *T. gondii*.

These findings underscore the importance of testing compounds in biologically relevant models and support the potential of 6-nitrocoumarin-3-thiosemicarbazone hybrids as lead-like structures. Although their potency is still moderate, their selectivity and activity in both *in vitro* and *ex vivo* models justify further structure-based optimization efforts to enhance intracellular efficacy and assess pharmacokinetic and resistance profiles in optimized derivatives.

## MATERIALS AND METHODS

### General procedures

All common laboratory chemicals were purchased from commercial sources and used without further purification. 3-Acetyl-4-hydroxycoumarin was synthesized according to a literature procedure (82). Anton Paar Monowave 50 conventionally heated reactor, provided with sealed vessel heating of 10 mL tubes with 250°C maximum temperature and 20 bar pressures by applying conductive heating principles, was used for carrying out the Sealed Vessel reactions (SVR). All NMR spectra were recorded in DMSO-d_6_ at 300 K on a Bruker Avance 400 MHz spectrometer equipped with a 5 mm QXI probe and on an Avance III 400 MHz spectrometer equipped with a 5 mm BBOF plus probe. Chemical shifts (δ) are expressed in ppm using tetramethylsilane (TMS) as internal reference, and multiplicities are indicated as s (singlet), d (doublet), t (triplet), q (quartet), m (multiplet), and b (broad). The assignments of ^1^H and ^13^C-NMR resonances were made based on a combination of ^1^H, ^1^H-COSY, ^1^H, ^13^C-HSQC, and ^1^H, ^13^C-HMBC spectra. C, H, N, and S analyses were carried out with a Thermo Scientific Flash 2000 elemental analyzer. The FTIR spectra (4,000–400 cm^–1^) were measured as KBr pellets (1%) with a Shimadzu IRPrestige-21 instrument. Electrochemical studies were carried out on a Metrohm Autolab instrument model PGSTAT204, using dimethyl sulfoxide (DMSO) as the solvent (1 mM solutions), tetrabutylammonium perchlorate (0.1 M TBAP) as the supporting electrolyte, a hanging drop mercury electrode (HDME) as the working electrode, Ag/AgCl (3M KCl) as the reference electrode and a graphite rod as the counter electrode. All measurements were performed after bubbling with nitrogen (N_2_) for 10 minutes. ESR studies were conducted on a Bruker ECS 106 X-band (9.85 GHz) spectrometer with a rectangular cavity and a field modulation of 50 kHz. *In situ* reduction of the compounds under study was carried out at potentials determined by cyclic voltammetry (CV) to generate radical species. DMSO was used as the solvent, TBAP as the supporting electrolyte, and a platinum wire as the working electrode. Lipophilicity was studied by reversed-phase TLC experiments. Precoated TLC plates SIL RP-18W/UV254 were used and eluted with DMSO/physiological serum (70:30, vol/vol). Stock solutions were prepared in pure DMSO (Aldrich) prior to use. The plates were developed in a closed chromatographic tank, dried, and the spots were located under UV light. The Rf values were averaged from three determinations and converted into R_M_ values via the relationship: R_M_ = log [(1/Rf)−1] (83).

### Synthesis of 6-nitro-coumarin-3-thiosemicarbazone

#### Synthesis of 3-acetyl-4-hydroxy-6-nitro-2H-chromen-2-one (C1)

Compound **C1** was obtained using a modified procedure based on a reported procedure (46). To a stirred solution of KNO_3_ (1 mmol) in H_2_SO_4_ conc. (1 mmol, 4 mL) was added portion-wise 3-acetyl-4-hydroxy-2H-chromen-2-one (3.5 mmol, 714 mg). The reaction mixture was stirred for 1 h at 0°C and the added ice (50 g) until precipitation of a white solid. The solid is filtered, washed with cold water (50 mL), and dried under vacuum for 48 h to give compound **C1** (448 mg, 53%) as a white solid: ^1^H NMR δ 8.69 (d, J = 2.7 Hz, ^1^H), 8.58 (dd, J = 9.1, 2.8 Hz, 1H), 7.67 (d, J = 9.1 Hz, 1H), 2.69 (s, 3H); ^13^C NMR δ: 204.99, 176.74, 158.55, 157.60, 143.64, 130.50, 121.05, 118.74, 115.96, 102.24, and 29.46.

#### Synthesis of 3-acetyl-6-nitro-2H-chromen-2-one (C2)

Compound **C2** was obtained using a modified procedure based on a reported procedure (47). In a sealed vessel, the reactor tube was dissolved 2-hydroxy-5-nitrobenzaldehyde (3.5 mmol, 585 mg) in EtOH (2 mL) and added with stirring ethyl acetoacetate (3.5 mmol, 446 mL) and piperidine (5% mol). The reaction mixture was heated to 50°C for 18 min. The obtained precipitate was filtered, washed with EtOH (2 × 3 mL), and dried for 48 h to yield compound C2 (408 mg, 50% yield) as a with solid: ^1^H NMR δ 8.94 (d, J = 2.8 Hz, 1H), 8.80 (s, 1H), 8.50 (dd, J = 9.1, 2.8 Hz, 1H), 7.68 (d, J = 9.1 Hz, 1H), 2.59 (s, 3H); ^13^C NMR δ: 194.70, 158.03, 157.47, 145.81, 143.75, 128.49, 126.45, 126.12, 118.54, 117.70, and 29.92.

### General procedure for the synthesis of compound 1–8

In a vessel reactor tube, the respective coumarin 3-acetyl-4-hydroxy-6-nitrocoumarin (**C1**) or 3-acetyl-6-nitrocoumarin (**C2**) (1 mmol) was poured and suspended in methanol (1 mL). Then the corresponding thiosemicarbazide (1 mmol) suspended in methanol (1 mL) was added to the mixture with stirring. After 1 minute p-TsOH (5%) was added and heated at 70°C for 12 minutes. The reaction was monitored by TLC until the starting material was consumed and cooled to room temperature. The mixture was filtered off, and the solid was washed with methanol (2 × 5 mL), diethylether (2 × 5 mL), and then air-dried. Compound **5** had been previously prepared through a different procedure (84).

(E)−2-(1-(4-hydroxy-6-nitro-2-oxo-2H-chromen-3-yl)ethylidene)hydrazine-1-carbothioamide **Compound 1—**yellow microcrystalline solid, yield = 68%. Elemental analysis: Calc. (%) for C_12_H_10_N_4_O_5_S C, 44.7; H, 3.1; N, 17.4, found: C, 44.9; H, 3.0; N, 17.5; ^1^H NMR, δ: 15.25 (s, 1H); 10.74 (s, 1H), 8.67 (d, *J* = 2.8 Hz, 1H), 8.46 (dd, *J* = 9.0, 2.9 Hz, 1H), 7.55 (d, *J* = 9.0 Hz, 1H), and 2.64 (s, 3H). ^13^C NMR, δ: 181.14, 176.48, 160.44, 156.77, 143.41, 128.72, 121.51, 119.89, 118.30, and 17.49.

(E)−2-(1-(4-hydroxy-6-nitro-2-oxo-2H-chromen-3-yl)ethylidene)-N-methylhydrazine-1-carbothioamide **Compound 2**—brown microcrystalline solid, yield = 79%. Elemental analysis: Calc. (%) for C_13_H_12_N_4_O_5_S: C, 46.4; H, 3.6; N, 16.7, found: C, 46.2; H, 3.7; N, 16.5. ^1^H NMR, δ: 15.06 (s, 1H), 10.59 (s, 1H), 8.67 (d, *J* = 2.9 Hz, 1H), 8.46 (dd, *J* = 9.0, 2.9 Hz, 1H), 7.55 (d, *J* = 9.1 Hz, 1H), 2.96 (s, 3H), and 2.64 (s, 3H). ^13^C NMR, δ: 181.32, 176.50, 160.46, 156.79, 143.42, 128.73, 121.54, 119.93, 118.31, 31.36, and 17.54.

(E)-N-ethyl-2-(1-(4-hydroxy-6-nitro-2-oxo-2H-chromen-3-yl)ethylidene)hydrazine-1-carbothioamide **Compound 3—**yellow microcrystalline solid, yield = 62%. Elemental analysis: Calc. (%) for (C_14_H_14_N_4_O_5_S): C, 48.0; H, 4.0; N, 16.0, found: C, 48.1; H, 4.1; N, 15.8. ^1^H NMR δ: 15.16 (s, 1H), 10.52 (s, 1H), 8.67 (d, *J* = 2.9 Hz, 1H), 8.51 (s, 1H), 8.46 (dd, *J* = 9.0, 2.9 Hz, 1H), 7.55 (d, *J* = 9,0 Hz, 1H), 3.60–3.40 (m, 2H), 2.64 (s, 3H), 1.14 (t, *J* = 7.2 Hz, 3H). ^13^C NMR δ: 180.20, 176.33, 160.46, 156.78, 143.41, 128.68, 121.51, 119.91, 118.29, 38.20, 17.53, and 13.94.

(E)−2-(1-(4-hydroxy-6-nitro-2-oxo-2H-chromen-3-yl)ethylidene)-N-phenylhydrazine-1-carbothioamide **Compound 4**—orange microcrystalline solid, yield = 74%. Elemental analysis: Calc. (%) for (C_18_H_14_N_4_O_5_S): C, 54.3; H, 3.5; N, 14.1, found: C, 54.1; H, 3.5; N, 14.3. ^1^H-NMR δ: 15.48 (s, 1H), 10.20 (s, 1H), 8.69 (d, *J* = 2.9 Hz, 1H), 8.45 (dd, *J* = 9.0, 2.9 Hz, 1H), 7.60–7.56 (m, 2H), 7.54 (d, *J* = 9.1 Hz, 1H), 7.37 (t, *J* = 7.9 Hz, 2H), 7.15 (t, *J* = 7.2 Hz, 1H), and 2.73 (s, 3H).^13^C NMR δ: 178.31, 175.99, 160.62, 156.81, 143.34, 139.05, 128.67, 128.41, 124.82, 122.95, 121.51, 120.20, 118.19, and 17.92.

(E)−2-(1-(6-nitro-2-oxo-2H-chromen-3-yl)ethylidene)hydrazine-1-carbothioamide **Compound 5**—yellow microcrystalline solid, yield = 86%. Elemental analysis: Calc. (%) for (C_12_H_10_N_4_O_4_S): C, 47.1; H, 3.3; N, 18.3, found: C, 47.2; H, 3.4; N, 18.5. ^1^H NMR δ: 10.56 (s, 1H), 8.68–8.65 (m, 2H), 8.51 (s, 1H), 8.42 (dd, *J* = 9.1, 2.8 Hz, 1H), 7.92 (s, 1H), 7.65 (d, *J* = 9.2 Hz, 1H), and 2.27 (s, 3H). ^13^C NMR δ: 179.36, 158.15, 156.91, 144.76, 143.63, 140.66, 127.37, 126.68, 124.68, 119.33, 117.52, 15.81.

(E)-N-methyl-2-(1-(6-nitro-2-oxo-2H-chromen-3-yl)ethylidene)hydrazine-1-carbothioamide **Compound 6**—yellow microcrystalline solid, yield = 28%. Elemental analysis: Calc. (%) for (C_13_H_12_N_4_O_4_S): C, 48.7; H, 3.8; N, 17.5, found: C, 48.5; H, 3.9; N, 16.9. ^1^H NMR δ: 10.57 (s, 1H), 8.72 (d, *J* = 2.7 Hz, 1H), 8.56 (s, 1H), 8.49–8.46 (m, 1H), 8.44 (dd, *J* = 9.1, 2.8 Hz, 1H), 7.67 (d, *J* = 9.1 Hz, 1H), 3.04 (d, *J* = 4.5 Hz, 3H), and 2.27 (s, 3H). ^13^C NMR δ: 178.94, 158.23, 156.89, 144.65, 143.70, 140.49, 127.79, 126.72, 124.65, 119.26, 117.60, 31.01, 15.85.

(E)-N-methyl-2-(1-(6-nitro-2-oxo-2H-chromen-3-yl)ethylidene)hydrazine-1-carbothioamide **Compound 7**—yellow microcrystalline solid, yield = 75%. Elemental analysis: Calc. (%) for (C_14_H_14_N_4_O_4_S): C, 50.3; H, 4.2; N, 16.8, found: C, 50.7; H, 4.4; N, 16.6. ^1^H NMR δ: 10.50 (s, 1H), 8.74 (d, *J* = 2.8 Hz, 1H), 8.53 (s, 1H), 8.48 (t, *J* = 5.8 Hz, 1H), 8.44 (dd, *J* = 9.1, 2.8 Hz, 1H), 7.67 (d, *J* = 9.1 Hz, 1H), 3.61 (p, *J* = 7.1 Hz, 2H), 2.26 (s, 3H), and 1.16 (t, *J* = 7.1 Hz, 3H). ^13^C NMR δ: 177.89, 158.14, 156.88, 144.75, 143.68, 140.46, 127.78, 126.72, 124.71, 119.26, 117.58, 38.44, 15.85, 14.39.

(E)−2-(1-(6-nitro-2-oxo-2H-chromen-3-yl)ethylidene)-N-phenylhydrazine-1-carbothioamide **Compound 8**—yellow microcrystalline solid, yield = 83%. Elemental analysis: Calc. (%) for (C_18_H_14_N_4_O_4_S): C, 56.5; H, 3.7; N, 14.6, found: C, 56.7; H, 3.9; N, 14.3. ^1^H NMR δ: 10.98 (s, 1H), 10.11 (s, 1H), 8.72 (d, *J* = 2.8, 1H) y 8.68 (s, 1H), 8.44 (dd, *J* = 9.1, 2.8 Hz, 1H), 7.67 (d, *J* = 9.1 Hz, 1H), 7.61 (m, 2H), 7.39 (t, *J* = 7.9 Hz, 2H), 7.22 (tt, *J* = 7.1, 1.1 Hz, 1H), and 2.35 (s, 3H). ^13^C NMR δ: 177.10, 158.03, 156.90, 145.48, 143.67, 140.95, 138.83, 128.27, 127.29, 126.81, 125.50, 125.28, 124.78, 119.28, 117.57, and 15.99.

### Biological studies

The trypomastigotes of the Dm28 strain of *T. cruzi*, tachyzoites of the RH strain of *T. gondii,* and mammalian cells were obtained from an in-house collection (Programa de Biología Integrativa, Facultad de Medicina, Universidad de Chile).

### Cell cultures

BeWo cells (ATCC CCL-98) were cultured in DMEM/F-12 (Dulbecco’s Modified Eagle Medium Nutrient Mixture F12) supplemented with 10% inactivated fetal bovine serum (FBSi) and 1% antibiotics (penicillin-streptomycin), incubated at 37°C in a humidified atmosphere with 5% CO_2_. Cells at semi-confluence were harvested by trypsinization and sedimented by centrifugation at 500 × *g* for 5 minutes at room temperature, re-suspended in DMEM-F12 medium, and counted with trypan blue using the JuLI FL Live Cell Analyzer device (85). EA.hy926 cells (ATCC CRL-2922) were cultured in DMEM high glucose (Dulbecco’s Modified Eagle Medium) supplemented with 10% FBSi and 1% antibiotics (penicillin-streptomycin). In this, and all cultures described below, cells were incubated at 37°C, with 5% CO_2_ and 98% relative humidity. VERO (ATCC CCL-81) cells were cultured in Roswell Park Memorial Institute 1640 medium (RPMI 1640), supplemented with 5% FBSi and 1% antibiotics (penicillin-streptomycin). In this, and all cultures described below, cells were incubated at 37°C, with 5% CO_2_ and 98% relative humidity. HFF (ATCC SCRC-1041) cells were cultured in DMEM supplemented with 10% FBSi and 1% antibiotics (penicillin-streptomycin). In this, and all cultures described below, cells were incubated at 37°C, with 5% CO_2_ and 98% relative humidity. LLC-MK2 (ATCC CCL-7) monkey kidney cells were cultured in DMEM, supplemented with 10% FBSi and 1% antibiotics (penicillin-streptomycin). In this, and all cultures described below, cells were incubated at 37°C, with 5% CO_2_ and 98% relative humidity. Cell passages were performed weekly.

### Parasite culture and harvesting *Trypanosoma cruzi*

VERO cells at semi-confluence were infected with trypomastigotes from the Dm28 strain and cultured in RPMI 1640 medium supplemented with 5% FBSi and 1% antibiotics (penicillin-streptomycin) at 37°C in a humidified atmosphere with 5% CO_2_. The parasites invaded the cells and replicated intracellularly as amastigotes. After 48 to 72 hours, amastigotes were allowed to differentiate back into trypomastigotes and released by cell lysis. Trypomastigotes were separated from cellular debris by centrifugation at 500 × *g* for 5 minutes and then recovered from the supernatant by centrifugation at 3,500 × *g* for 10 minutes at 4°C, re-suspended in RPMI 1640 medium supplemented (5% FBS and 1% antibiotics), and quantified using a Neubauer chamber (68).

The recombinant Tulahuen β-gal strain, expressing β-galactosidase, was employed (86). Trypomastigotes were obtained by infecting LLC-MK2 cells monolayers in 75 cm^2^ culture flasks. Cells were infected for 48 hours with 1.5 × 10^5^ trypomastigotes in 10 mL DMEM supplemented with 2% FBSi and 1% antibiotics (penicillin/streptomycin) and incubated. After 48 hours, the cell monolayer was washed with sterile PBS, and the medium was replaced. Trypomastigotes were collected starting at day 5 post-infection, pelleted by centrifugation, and washed 2× in sterile PBS. The number of parasites was determined microscopically, using a hemocytometer.

### Toxoplasma gondii

HFF cells at semi-confluence were infected with tachyzoites from the RH strain and cultured in DMEM medium supplemented with 1% FBSi and 1% antibiotics at 37°C in a humidified atmosphere with 5% CO_2_. After 48 hours, tachyzoites in the supernatant were collected. Parasites in the culture medium were purified by filtration through a 3 µm polycarbonate membrane (IsoporeTM Membrane Filters, Merck Millipore), eliminating cellular debris. Then, tachyzoites were recovered by centrifugation at 3,500 × *g* for 5 minutes at room temperature, re-suspended in DMEM medium supplemented (1% FBSi and 1% antibiotics), and quantified using a Neubauer chamber (87).

### Cytotoxicity on cells

BeWo or EA.hy926 cells were seeded in 96-well plates, with 5 × 10^4^ cells per well, and incubated with the compounds chosen as most active against *T. cruzi* and *T. gondii* (previously dissolved in DMSO) at concentrations ranging from 17 μM to 400 µM for 24 hours at 37°C in a humid atmosphere with 5% CO_2_. Triton X-100 was used as a positive control, and untreated cells served as a negative control. In addition, the maximum concentration of DMSO was used as a control for the method. After the incubation period, cells were washed with PBS, and 100 µL of DMEM-F12 medium with resazurin to a final concentration of 3 mM was added to each well, followed by incubation for 3 hours at 37°C in a humidified atmosphere with 5% CO_2_. After incubation, measurements were taken using a microplate reader (Thermo Scientific Varioskan Flash) at excitation wavelengths of 560 nm and emission wavelengths of 590 nm. The obtained values were expressed as IC_50_(88).

### Activity on *T. cruzi* trypomastigotes and *T. gondii* tachyzoites

Parasites at 10^7^ parasites/mL concentration were incubated for 24 hours with the compounds under investigation (previously dissolved in DMSO) at a concentration of 100 µM at 37°C in a humid atmosphere with 5% CO_2,_ DMSO concentration did not exceed 0.5% vol/vol. NFX was used as a positive control for *T. cruzi*, and PMN was used for *T. gondii*. Untreated parasites served as a negative control. In addition, considering that the compounds were dissolved in DMSO, a control condition with the maximum possible DMSO concentration was used to verify that this solvent alone has no antiparasitic or cytotoxic activity. Subsequently, parasites with their respective conditions were transferred to a 96-well white fluorescence reading plate, adding resazurin at a final concentration of 3 mM with 0.8 mM phenazine. The plate was incubated for 3 hours at 37°C in a humidified atmosphere with 5% CO_2_, and fluorescence measurements were taken using a microplate reader (Thermo Scientific Varioskan Flash) at excitation wavelengths of 560 nm and emission wavelengths of 590 nm. Once the most active compound against the parasites was identified, the IC_50_ value, corresponding to the inhibitory concentration of 50% of the parasite population, was determined (38, 89).

### Effect on intracellular parasite

LLC-MK2 cells (2 × 10^4^/well), suspended in 200 µL DMEM with 10% FBSi and 1% antibiotics (penicillin-streptomycin), were placed in each well of a 96-well plate and incubated for 24 hours. Subsequently, the culture medium was removed, and cell monolayers were infected by placing five trypomastigotes per cell in 200 µL of DMEM 2 for 24 hours under the same incubation conditions. After infection, the culture medium was removed, and cells were washed 4 times with 1× sterile PBS. Finally, 10 twofold serial dilutions (200 µM to 0.39 µM concentration range) of each compound, in DMEM 2 without phenol red, were placed in duplicate wells in a 96-well plate and incubated for 72 hours. Subsequently, inhibition of intracellular parasite multiplication was measured as in Bettiol et al. (90): 50 µL of a solution containing 100 µM chlorophenol red β-D-galactopyranoside (CPRG) and Triton (0.5%) in PBS was added to each well. Absorbance at 600 nm was read 4 hours later, in a multimodal GloMax microplate reader (Promega).

VERO cells (5 × 10^4^/well), suspended in 200 µL RPMI with 5% FBS and 1% antibiotics (penicillin-streptomycin), were placed in each well of a 96-well plate and incubated for 24 hours. Subsequently, the culture medium was removed, and cell monolayers were infected by placing one tachyzoite per cell in 200 µL of RPMI for 24 hours under the same incubation conditions. After infection, the culture medium was removed, and the cells were washed with sterile PBS. Then, concentrations of each compound (200 µM to 0.39 µM) in RPMI were placed in triplicate wells in a white 96-well plate and incubated for 24 hours. Finally, the cells were washed with PBS to remove the supernatant, fixed with 90% cold methanol, washed with PBS containing 0.05% Tween, and blocked using 3% BSA for 1 hour at 37°C. Then, they were incubated with the primary monoclonal antibody *Toxoplasma gondii* (TP3): sc-52255 at a dilution of 1:500 with 1% BSA overnight at 4°C. Afterward, the samples were incubated with the FITC anti-mouse IgG secondary antibody at a dilution of 1:100 with 1% BSA for 1 hour at 37°C. Finally, they were washed with PBS containing 0.05% and maintained in PBS. Fluorescence measurements were taken using a microplate reader (Thermo Scientific Varioskan Flash) at excitation wavelengths of 488 nm and emission wavelengths of 519 nm (see Fig. S8 at https://doi.org/10.5281/zenodo.15694236).

For the microscopic analysis of BeWo cells infected with *T. cruzi*, the cells were washed with PBS to remove the supernatant, fixed with 90% cold methanol, rewashed with PBS, and incubated for 5 minutes with 1 µg/mL—1 4,6-diamidino-2-phenylindole (DAPI) (Molecular Probes). To analyze BeWo cells infected with *T. gondii*, cells were fixed with 90% cold methanol, washed with PBS containing 0.05% Tween, and blocked using 3% BSA for 1 hour at 37°C. Then, they were incubated with the primary monoclonal antibody *Toxoplasma gondii* (TP3): sc-52255 at a dilution of 1:500 with 1% BSA overnight at 4°C. Afterward, the samples were incubated with the FITC anti-mouse IgG secondary antibody at a dilution of 1:100 with 1% BSA for 1 hour at 37°C. Finally, they were washed with PBS containing 0.05% Tween and incubated for 5 minutes with DAPI. Subsequently, the sections were mounted in Vectashield (ScyTek ACA). Sections for the analysis of cell infection by *T. cruzi* and *T. gondii* were observed under an epifluorescence microscope (Motic BA 310), and images were captured with a Motic 5 camera. *T. cruzi* amastigotes were identified by their morphology, including nuclear size and the presence of a kinetoplast, and were analyzed and quantified using ImageJ. At least 500 cells per condition were analyzed, and infected and non-infected cells were counted for every 100 cells (35, 70).

### Intracellular generation of ROS

ROS detection was performed using the 2′,7′-dichlorodihydrofluorescein diacetate (DCFH_2_-DA) method (45). For the assay, *Dm28c* trypomastigotes and tachyzoites were seeded in 96-well plates at a density of 10⁶ parasites/mL. In contrast, BeWo cells were seeded at 5 × 10⁴ cells per well, using the previously described culture medium.

After incubation with 20 µM DCFH_2_-DA for 15 minutes at 37°C, the parasites were centrifuged at 3,500 × *g*, washed twice with PBS (pH 7.4), and subsequently transferred to Nunc 96-well fluorescence plates. Meanwhile, after 24 hours of incubation at 37°C and 5% CO₂, the culture medium of BeWo cells was replaced, and 20 µM DCFH_2_-DA solution was added, followed by a 15 minute incubation at 37°C. The cells were then washed with PBS at pH 7.4. As a positive control, 20 µM menadione was used, while the negative control consisted of culture medium without compounds.

Subsequently, the most active compounds were added at the IC_50_ concentration, and fluorescence (excitation: 488 nm, emission: 528 nm) was recorded for 40 minutes using a Thermo Scientific Varioskan Lux microplate reader. Over time, the area under the fluorescence increase curve was determined using Origin 8 software (version 9.2), with normalization relative to the control. The results correspond to three independent experiments' mean ± standard deviation (SD).

### Electron spin resonance studies in parasite media

The free radical production capacity of the most active 6-nitrocoumarin-3-thiosemicarbazone hybrids against each parasite was evaluated using electron spin resonance (ESR) spectroscopy, with 5,5-dimethyl-1-pyrroline-N-oxide (DMPO) as a spin-trapping agent (41, 45).

Each tested hybrid was dissolved in spectroscopy-grade DMSO at an approximate concentration of 1 mM and added to a mixture containing *Trypanosoma cruzi* (Dm28c strain) or *Toxoplasma gondii* at a density of 50 × 10⁶ trypomastigotes or tachyzoites per mL, along with DMPO at a final concentration of 250 mM. Menadione (20 µM) was used as a positive control.

The mixture was transferred to a 50 µL capillary, and ESR spectra were recorded in the X-band (9.85 GHz) using a Bruker ECS 106 spectrometer with a rectangular cavity and 50 kHz field modulation. All spectra were acquired on the same scale after 15 scans.

### Obtention of HPE

Term human placentas were obtained from uncomplicated pregnancies via scheduled cesarean sections. Each patient provided informed consent for the experimental use of the placenta, and the study was approved by the ethics committee of Hospital San José, Servicio Salud Metropolitano Norte (N° 013/2022). Exclusion criteria for placental use included significant fetal anomalies, placental tumors, intrauterine infection, positive serology for Chagas disease, obstetric pathology, and any other maternal disease. Donor patients tested negative for *T. gondii* IgG/IgM antibodies. Organs were collected in sterile, cold PBS and processed within 30 minutes after delivery. Maternal and fetal surfaces of each placenta were discarded, and the villous tissue from the central part of the placental cotyledons was isolated. Isolated chorionic villi were washed with PBS to remove the blood and cut into approximately 0.5 cm^3^ pieces, incubated in RPMI medium supplemented with 5% FBS and 1% antibiotics (penicillin-streptomycin) at 37°C in a humid atmosphere with 5% CO_2_ overnight (79). All the experiments were performed in triplicate in at least three different placentas.

### Effect of compounds on infection in *ex vivo* and *in vitro* model

Cells or HPE were exposed to parasites. Cells were revealed in a 1:1 relation (cell: parasite), and HPE was exposed to 1 × 10^5^ parasites/mL for 24 hours. The following treatment conditions were used in both infection models. (i) Parasites used during infection were co-incubated for 24 hours with the most active nitrocoumarin-thiosemicarbazone derivative against each parasite at the previously obtained IC_50_ concentration. (ii) A post-infection treatment condition was applied by treating cells or HPE infected for 24 hours, removing parasites from the medium, and adding the most active nitrocoumarin-thiosemicarbazone derivative for the corresponding parasite at the IC_50_ concentration for 24 hours. Untreated cells or HPE served as a negative control, and the compounds’ activity was compared with the reference drugs (NFX and PMN). This approach allowed the observation of the effects of the most active compounds on the infection of BeWo cells and HPE against each of the parasites for both the infective and intracellular proliferative forms.

### DNA amplification by real-time PCR

For the parasitic DNA load in the *in vitro* infection model, BeWo cells were seeded in 6-well plates with 5 × 10^5^ cells and were allowed to adhere overnight at 37°C in a humidified atmosphere with 5% CO_2_. Subsequently, cells were exposed to parasites at a 1:1 ratio (cell: parasite) for 24 hours. Finally, the cells were washed with PBS and harvested using trypsin. For the parasitic DNA load in the *ex vivo* infection model, HPE was incubated in 24-well plates and exposed to parasites at a concentration of 10^5^ parasites/mL for 24 hours. Finally, they were washed with PBS and homogenized using the ultraturrax homogenizer OMNI TipTM. Genomic DNA was extracted from BeWo cells or HPE using the Wizard Genomic DNA Purification Kit (Promega, USA) following the manufacturer’s instructions. The resulting DNA was quantified using a μDrop Plate DNA quantification system on the Varioskan Flash multimode reader (Thermo Scientific, USA). For the amplification of human DNA and parasitic DNA, three pairs of primers specific for human GAPDH (hGADPH), *T. cruzi* satellite DNA, and *T. gondii* B1 gene DNA were used (Table 7). Each reaction mixture contained 0.5 µL of 10 nM of each primer (Forward and reverse), 1 ng of DNA from *T. cruzi* samples, 10 ng of DNA from *T. gondii* samples, 10 µL of SensiFASTTM SYBR Hi-ROX kit (Bioline, Heidelberg, Baden Württemberg, Germany), and ultrapure water (Sigma-Aldrich, St. Louis, MO, USA) for a total of 20 µL.

**TABLE 7:** Primers for the identification and amplification of *T. cruzi* DNA (Satellite DNA), *T. gondii* DNA (B1 Gene), and human DNA of the GAPDH gene as an endogenous loading control^*a*^

qPCR primer	Forward sequence	Reverse sequence	Amplicon length (bp)
*T. cruzi*	GCTCTTGCCCCACAMGGTGC	CAAGCAGCGGATAGTTCAG	182
*T. gondii*	AGCGTTCGTCCTCAACTATCGATTG	TCCCCTCTGCTCGCGAAAAGT	98
hGAPDH	TGATGCGTGTACAAGCGTTTT	ACATGGTATTCACCACCCCACTAT	97

^*a*^ Primer sequences are in the 5′ to 3′ direction.

Amplification was carried out using the CFX96 Touch Deep Well Real-Time PCR Detection System (Biorad, California, USA) with the following cycling program: initial denaturation at 95°C for 3 min, followed by 40 cycles of denaturation at 95°C for 5 s, annealing at 60°C for 30 s, and a dissociation stage ranging from 60°C to 95°C. The relative quantification analysis of the results was expressed as RQ values using the comparative control method (∆∆Ct) (80).

### Histological and histochemical techniques

The HPE was fixed in 10% formaldehyde in 0.1 M phosphate buffer (pH 7.3) for 24 h, dehydrated in alcohol, clarified in xylene, embedded in paraffin, and sectioned at 3 µm. Paraffin histological sections were stained with hematoxylin-eosin for routine histological analysis, Picro Sirius red, and trichromic Masson for collagen histochemistry. Sections for the histological analysis were observed under an optical microscope (Leica DM500) and images were captured with a Leica ICC50 W camera (7, 91).

### ADME properties

The pharmacokinetic properties of the synthesized 6-nitrocoumarin-3-thiosemicarbazone derivatives were evaluated using SwissADME (http://www.swissadme.ch, accessed on February 12, 2025). The analysis included predictions of absorption, distribution, metabolism, and excretion (ADME), as well as an evaluation of drug-likeness based on Lipinski’s Rule of Five (56).

Lipophilicity (logP) was determined using the iLOGP model, which provides a theoretical estimation of the compounds’ permeability across biological membranes. Aqueous solubility was predicted using SwissADME’s computational algorithms, classifying the compounds as highly soluble, moderately soluble, or poorly soluble. The topological polar surface area (TPSA) was calculated to estimate intestinal absorption and blood-brain barrier (BBB) permeability.

Metabolic stability was assessed by predicting potential interactions with major cytochrome P450 (CYP) isoforms, including CYP1A2, CYP2C19, CYP2C9, CYP2D6, and CYP3A4, to identify possible metabolic liabilities and drug-drug interactions. In addition, oral bioavailability was evaluated through the bioavailability radar, which provides a graphical representation of key pharmacokinetic parameters.

Molecular structures were input into SwissADME using the SMILES format, enabling the generation of relevant descriptors for analysis. The computational results were compared with experimental data on lipophilicity (RM values) and antiparasitic activity, allowing for a better understanding of the relationship between physicochemical properties and biological efficacy.

### Statistics

Parasites and untreated cells were used as the negative control, corresponding to 100% cell viability. The obtained data were expressed as the mean ± standard deviation for each condition and were processed using GraphPad Prism 8 software. For the study of the compound series against parasites and cells, statistical significance was analyzed using a one-way ANOVA test with Dunnett’s post-test, considering values with *P* ≤ 0.05 as statistically significant.
